# The Japanese Lung Cancer Society Guideline for non-small cell lung cancer, stage IV

**DOI:** 10.1007/s10147-019-01431-z

**Published:** 2019-05-02

**Authors:** Hiroaki Akamatsu, Kiichiro Ninomiya, Hirotsugu Kenmotsu, Masahiro Morise, Haruko Daga, Yasushi Goto, Toshiyuki Kozuki, Satoru Miura, Takaaki Sasaki, Akihiro Tamiya, Shunsuke Teraoka, Yukari Tsubata, Hiroshige Yoshioka, Yoshihiro Hattori, Chiyo K. Imamura, Yuki Katsuya, Reiko Matsui, Yuji Minegishi, Hidenori Mizugaki, Kaname Nosaki, Yusuke Okuma, Setsuko Sakamoto, Takashi Sone, Kentaro Tanaka, Shigeki Umemura, Takeharu Yamanaka, Shinsuke Amano, Kazuo Hasegawa, Satoshi Morita, Kazuko Nakajima, Makoto Maemondo, Takashi Seto, Nobuyuki Yamamoto

**Affiliations:** 1 Internal Medicine III, Wakayama Medical University, Wakayama, Japan; 2 Department of Hematology, Oncology and Respiratory Medicine, Okayama University Graduate School of Medicine, Dentistry and Pharmaceutical Sciences, Okayama, Japan; 3 Division of Thoracic Oncology, Shizuoka Cancer Center, Shizuoka, Japan; 4 Department of Respiratory Medicine, Nagoya University Graduate School of Medicine, Aichi, Japan; 5 Osaka City General Hospital, Osaka, Japan; 6 Department of Thoracic Oncology, National Cancer Center Hospital, Tokyo, Japan; 7 Clinical Research Center, Department of Thoracic Oncology and Medicine, National Hospital Organization Shikoku Cancer Center, Ehime, Japan; 8 Department of Internal Medicine, Niigata Cancer Center Hospital, Niigata, Japan; 9 Respiratory Center, Asahikawa Medical University Hospital, Hokkaido, Japan; 10 National Hospital Organization Kinki-chuo Chest Medical Center, Osaka, Japan; 11 Department of Internal Medicine, Division of Medical Oncology and Respiratory Medicine, Shimane University Faculty of Medicine, Shimane, Japan; 12 Department of Thoracic Oncology, Kansai Medical University Hospital, Osaka, Japan; 13 Department of Thoracic Oncology, Hyogo Cancer Center, Hyogo, Japan; 14 Advanced Cancer Translational Research Institute, Showa University, Tokyo, Japan; 15 Department of Surgery, University of California San Diego, California, USA; 16 National Cancer Center Hospital East, Chiba, Japan; 17 Department of Pulmonary Medicine and Oncology, Graduate School of Medicine, Nippon Medical School, Tokyo, Japan; 18 First Department of Medicine, Hokkaido University Hospital, Hokkaido, Japan; 19 Thoracic Oncology, National Hospital Organization Kyushu Cancer Center, Fukuoka, Japan; 20 Department of Thoracic Oncology and Respiratory Medicine, Tokyo Metropolitan Cancer and Infectious diseases Center Komagome Hospital, Tokyo, Japan; 21 Kyushu University Hospital, Fukuoka, Japan; 22 Regional Respiratory Symptomatology, Kanazawa University Graduate School of Medical Science, Kanazawa, Japan; 23 Research Institute for Diseases of the Chest, Graduate School of Medical Sciences, Kyushu University, Fukuoka, Japan; 24 Department of Thoracic Oncology, National Cancer Center Hospital East, Chiba, Japan; 25 Department of Biostatistics, Yokohama City University School of Medicine, Kanagawa, Japan; 26 Japan Federation of Cancer Patient Groups, Tokyo, Japan; 27 Japan Lung Cancer Alliance, Tokyo, Japan; 28 Department of Biomedical Statistics and Bioinformatics, Kyoto University Graduate School of Medicine, Kyoto, Japan; 29 Shizuoka Cancer Center, Shizuoka, Japan; 30 Division of Pulmonary Medicine, Allergry and Rheumatology, Department of Internal Medicine, Iwate Medical University School of Medicine, Iwate, Japan

**Keywords:** Non-small cell lung cancer, Chemotherapy, Kinase inhibitor, Programed cell death-1 inhibitor, Programed death-ligand 1 inhibitor, Guideline

## Abstract

According to rapid development of chemotherapy in advanced non-small cell lung cancer (NSCLC), the Japan Lung Cancer Society has been updated its own guideline annually since 2010. In this latest version, all of the procedure was carried out in accordance with grading of recommendations assessment, development and evaluation (GRADE) system. It includes comprehensive literature search, systematic review, and determination of the recommendation by multidisciplinary expert panel which consisted of medical doctors, pharmacists, nurses, statisticians, and patients from patient advocacy group. Recently, we have had various types of chemotherapeutic drugs like kinase inhibitors or immune-checkpoint inhibitors. Thus, the guideline proposes to categorize patients into three entities: (1) driver oncogene-positive, (2) PD-L1 ≥ 50%, and (3) others. Based on this subgroup, 31 clinical questions were described. We believe that this attempt enables clinicians to choose appropriate treatment easier. Here, we report an English version of the Japan Lung Cancer Society Guidelines 2018 for NSCLC, stages IV.

## Literature search

We performed a comprehensive literature search using PubMed. Only English written literature was collected, and randomized trials or meta-analyses were included. Several reports that did not meet these criteria were adopted, but only when all committee members considered them as clinically important in terms of safety or efficacy. Committee members performed literature searches from Dec. 2004 to Jun. 2013 using the keywords “lung cancer and chemotherapy.” After Jul. 2013, the Japan Medical Library Association kindly supported us and performed literature searches using a method devised by their own specialists. In their searches, papers published until Dec 31, 2017 were collected using text from clinical questions (CQs) as keywords. Several conference presentations were adopted regardless of their date when they were considered to provide huge influence on our daily practice.

## Process of guideline development

Guideline committee (expert panel) consisted of medical doctors, pharmacists, nurses, statisticians, and patients from patient advocacy group. Initially, guideline committee members developed clinical questions (CQs) and collected evidences as described above. Evidences were systematically reviewed for every CQ and committee members decided the strength of evidence (Table 1). Considering this strength of evidence and other factors (i.e. risk–benefit balance and social values), we determined the final recommendation. In this guideline, we have two levels of recommendations (1; strong or 2; weak) in two directions (to do or not to do) (Table 2). Recommendation was accepted when at least 60% of expert panel members reached an agreement. Whole process of developing guideline was carried out in accordance with Grading of Recommendations Assessment, Development and Evaluation (GRADE) system (http://www.gradeworkinggroup.org/).

**Table 1:** Strength of evidence

Level	Strength	Example
A	High	Several high-quality studies with consistent results
B	Moderate	One high-quality study
C	Low	Studies with severe limitations
D	Very low	Studies with very severe limitations or expert opinion

**Table 2:** Four types of recommendations

Recommendation level	Direction
1 (strong)	To do (= recommend to do)
2 (weak)	To do (= suggest to do)
1 (strong)	Not to do (= recommend not to do)
2 (weak)	Not to do (= suggest not to do)

## Outline

Cytotoxic chemotherapy has long been a mainstream component in the treatment of stage IV NSCLC. A meta-analysis showed that cytotoxic chemotherapy significantly prolonged overall survival (OS) compared with best supportive care (BSC) [1]. Cytotoxic chemotherapy accounted for 9% increase in 1-year survival (from 20 to 29%), or 1.5-month prolongation of OS. Another analysis, using third-generation cytotoxic chemotherapy, showed that monotherapy led to about 7% improvement in 1-year survival compared with BSC [2]. Regarding toxicity, another meta-analysis regarding NSCLC showed that treatment-related deaths with cytotoxic chemotherapy comprised 1.26%, and consisted of febrile neutropenia, cardiovascular events, and pulmonary toxicities [3]. Third-generation cytotoxic chemotherapy alone also showed better quality-of-life (QOL) scores than BSC [4]. Platinum agent plus third-generation cytotoxic chemotherapy showed similar QOL compared with third-generation cytotoxic chemotherapy alone, while progression-free survival (PFS) and overall survival (OS) were better in combination chemotherapy in a phase III trial [5].

Since the 2000s, novel agents such as molecular-targeted drugs and immune-checkpoint inhibitors (ICIs) have been demonstrating better outcomes compared with cytotoxic chemotherapy.

Most of the molecular-targeted drugs used in the treatment of NSCLC inhibit specific targets that induce cancer evolution [so-called “oncogenic drivers (i.e., *EGFR mutation, ALK translocation, ROS1 translocation*, and *BRAF mutation*)”]. Among those patients who harbor such oncogenic drivers and have good PS, these inhibitors demonstrated both ORR and PFS improvement. Phase III trials were conducted in *EGFR- and ALK-altered* patients, and kinase inhibitors have been shown to be more effective compared with cytotoxic chemotherapy [6–12]. For other types of genetic alterations, phase III trials have not been conducted, due to their rarity. Instead, similar efficacy results were observed in phase II trials or subset analyses of phase III trials. Molecular-targeted drugs usually showed milder toxicity than cytotoxic chemotherapy [13–16]. It is also important that they showed beneficial results in patients with poor PS in relatively small, but prospective studies [17, 18].

Since 2015, ICIs, which has a novel mode of action compared with other chemotherapeutic drugs, have been approved for administration in Japan. ICIs target immune-checkpoint molecules such as PD-1/L1, which are negative regulators in tumor immunity. A phase III trial, KEYNOTE-024, compared pembrolizumab (PD-1 inhibitor) with platinum-doublet chemotherapy in *EGFR*/*ALK-negative*, advanced NSCLC patients with tumor positive for PD-L1 ≥ 50% [19]. Pembrolizumab monotherapy demonstrated significant improvement of ORR, PFS, and OS, with tolerability. In addition to this, several phase III studies showed higher efficacy of platinum-based chemotherapy plus ICI than platinum-based chemotherapy alone in advanced NSCLC [20–23].

**Fig. 1:**
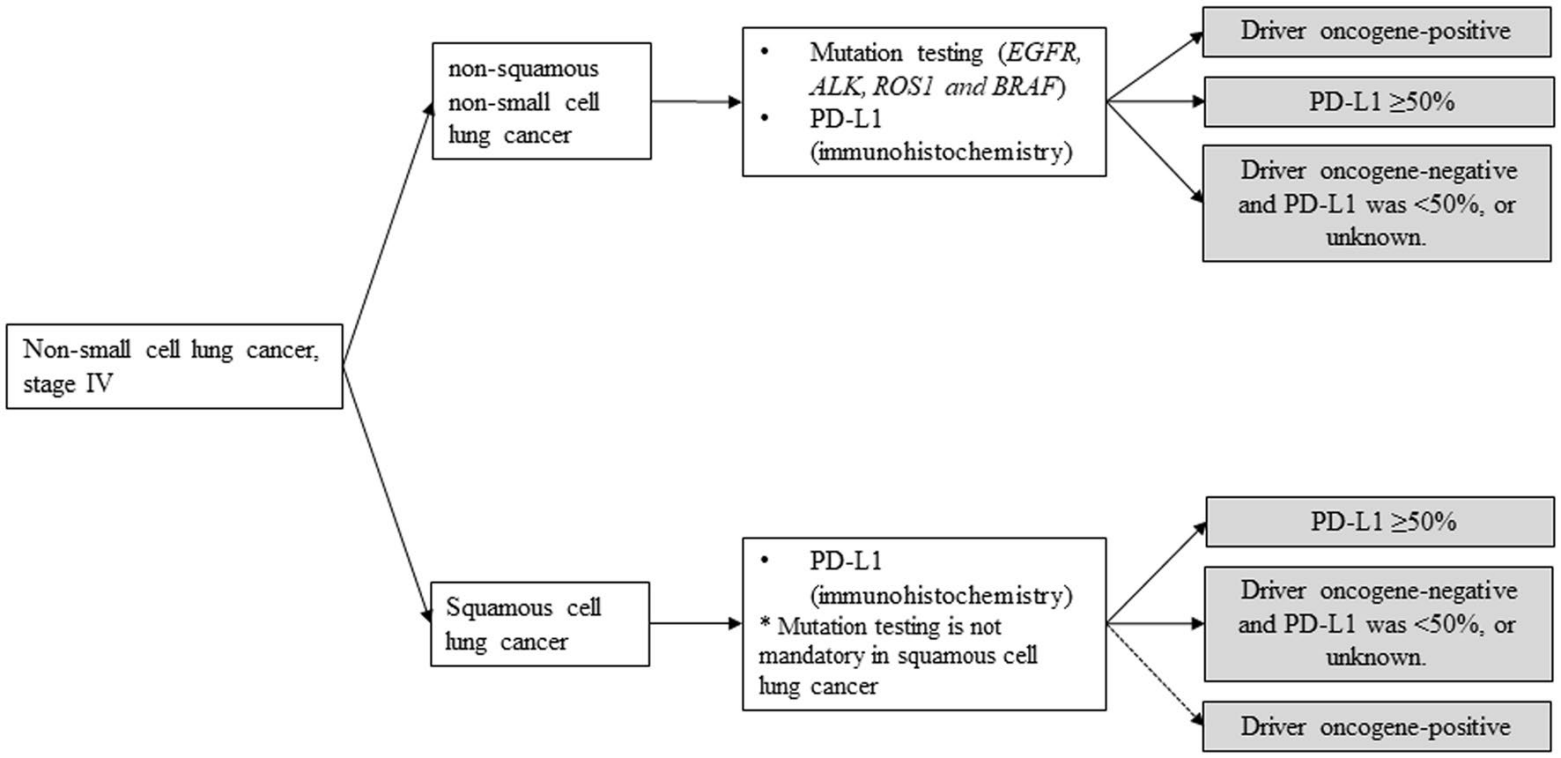
After diagnosis, patients will be categorized into three subgroups. *EGFR* epidermal growth factor receptor, *ALK* anaplastic lymphoma kinase, *PD-L1* programed death-ligand 1

**Fig. 2:**
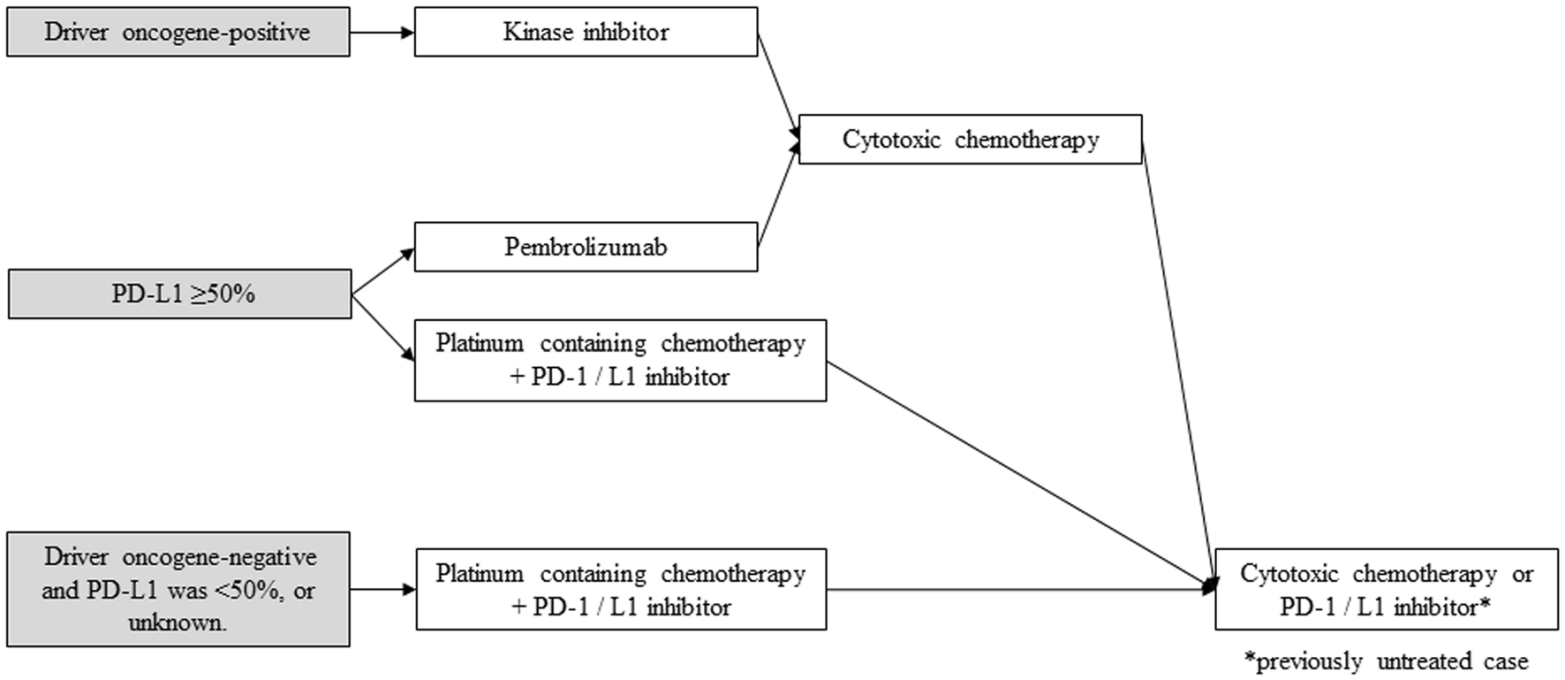
Treatment strategy of each subgroups in NSCLC, stage IV. *PD-1* programed cell death-1, *PD-L1* programed death-ligand 1

In summary, chemotherapy (cytotoxic chemotherapy, molecular-targeted drugs, and ICIs) demonstrated prolongation of OS and improvement of QOL compared with BSC among advanced NSCLC patients with good PS. To determine optimal treatment regimen, it is essential to check appropriate biomarkers such as driver oncogenes for molecular-targeted drugs and PD-L1 status for ICIs before their use. Therefore, while diagnosing advanced NSCLC patients, it is also important to categorize them into three entities: (1) driver oncogene-positive, (2) PD-L1 ≥ 50%, and (3) others (Fig. 1). Treatment strategy of each of these subgroups is described below (Fig. 2).

1. *Driver oncogene-positive* [CQ 1–17]

As previously described, kinase inhibitors for their specific driver oncogenes demonstrated improvement of ORR and PFS. OS was not improved, because most of the patients in standard arm received kinase inhibitors after progression. A large observational study in *EGFR-mutated* patients demonstrated that PFS with erlotinib did not differ, regardless of treatment line [24]. A firm conclusion regarding the treatment sequence between kinase inhibitors and cytotoxic chemotherapy cannot be drawn. However, a prospective observational study in the U.S. analyzed 10 genes in 733 patients, and oncogenic drivers were detected among 466 patients (64%). The study also showed that the patients who had oncogenic drivers and received kinase inhibitors lived longer than those who had oncogenic drivers, but did not receive inhibitors (3.5 years versus 2.4 years, propensity score-adjusted hazard ratio: 0.69, 95% CI: 0.53–0.90, *p* = 0.006) [25]. This evidence supports the notion that patients with driver oncogene should not lose the chance of receiving kinase inhibitors, and this guideline recommends that clinicians should administer kinase inhibitors prior to cytotoxic chemotherapy in this subgroup. In the second-line setting, cytotoxic chemotherapy is recommended in accordance with the patient’s general condition: CQ 20–23.

Regarding ICI for patients with oncogenic drivers, refer to CQ 5 and 6. Regarding squamous cell carcinoma with oncogenic drivers, refer to CQ 17.

2. *PD-L1 ≥ 50%* [CQ 18 and 19]

As data suggest that this subgroup can receive much benefit from ICI, pembrolizumab monotherapy is recommended. Similarly, platinum-based chemotherapy plus PD-1/L1 inhibitor is recommended. In the second-line setting, cytotoxic chemotherapy is recommended in accordance with the patient’s general condition.

3. *Others (driver oncogene-negative and PD-L1 < 50%, or unknown)* [CQ 20–31]

No molecular-targeted drugs or ICIs showed benefit compared with cytotoxic chemotherapy in this population. Conventional chemotherapy is the standard of care in accordance with the patient’s PS, age, and histology, while platinum-based chemotherapy plus PD-1/L1 inhibitor is recommended when patients have good PS.

Note: Definition of the elderly.

In Japan, patients who are aged 70–75 years are considered as elderly. However, patients who are ≥ 75 years of age have historically been excluded from clinical trials in Japan. Recently, however, most participants of phase II and phase III trials in elderly patients have been ≥ 75 years of age. Based on these considerations, this guideline defines elderly patients as those who are ≥ 75 years of age.

**Table:** **Summary of clinical questions**

Driver oncogene-positive
**Treatment of driver oncogene-positive patients (non-squamous cell lung cancer)**
CQ 1. What is the optimal first-line treatment for patients who have driver oncogene with good PS (0–1)?
CQ 2. What is the optimal first-line treatment for patients who have driver oncogene with poor PS (2–4)?
CQ 3. What is the optimal first-line treatment for elderly (≥ 75 years) patients who have driver oncogene?
CQ 4. Is cytotoxic chemotherapy recommended for patients who have driver oncogene?
CQ 5. Is combination of cytotoxic chemotherapy and ICI recommended for patients who have driver oncogene?
CQ 6. Is ICI recommended for patients who have driver oncogene?
***EGFR-mutated*** **, non-squamous cell lung cancer**
*First-line treatment in patients who have EGFR mutation (exon 19 deletion or L858R mutation)*
CQ 7. What is the recommended first-line treatment in patients who have *EGFR mutation* (*exon 19 deletion* or *L858R mutation*) with PS of 0–1?
CQ 8. What is the recommended first-line treatment in patients who have *EGFR mutation* (*exon 19 deletion* or *L858R mutation*) with PS of 2?
CQ 9. What is the recommended first-line treatment in patients who have *EGFR mutation* (*exon 19 deletion* or *L858R mutation*) with PS of 3–4?
*First-line treatment of those patients who have EGFR mutation other than exon 19 deletion or L858R mutation*
CQ 10. What is the recommended first-line treatment in patients who have *EGFR mutation* other than *exon 19 deletion* or *L858R mutation*, with PS of 0–1?
*Second-line and further treatment of those patients who have EGFR mutation*
CQ 11. What is the recommended second-line treatment in patients with *EGFR T790M mutation* after progression of EGFR-TKIs?
***ALK rearranged*** **, non-squamous cell lung cancer**
*First-line treatment of those patients who have ALK rearrangement*
CQ 12. What is the recommended first-line treatment in patients who have *ALK rearrangement* with PS of 0–1?
CQ 13. What is the recommended first-line treatment in patients who have *ALK rearrangement* with PS of 2–4?
*Second-line treatment of those patients who have ALK rearrangement*
CQ 14. What is the recommended second-line treatment in patients with *ALK rearrangement* after progression of ALK-TKIs?
***ROS1 rearranged*** **, non-squamous cell lung cancer**
CQ 15. Is crizotinib a recommended treatment in patients who have *ROS1 rearrangement*?
***BRAF mutated*** **, non-squamous cell lung cancer**
CQ 16. Is dabrafenib plus trametinib a recommended treatment in patients who have *BRAF mutation*?
**Driver oncogene-positive, squamous cell lung cancer**
CQ 17. Is tyrosine-kinase inhibitor a recommended treatment in squamous cell lung cancer patients who have driver oncogene?

PD-L1 ≥ 50%
CQ 18. What is the recommended first-line treatment in patients with PS 0-1 and whose tumor is positive for PD-L1 ≥ 50%?
CQ 19. What is the recommended first-line treatment in patients with PS 2 and whose tumor is positive for PD-L1 ≥ 50%?

Driver oncogene-negative and PD-L1 < 50%, or unknown
**First-line treatment in patients who are driver oncogene-negative and PD-L1 < 50%, or unknown**
CQ 20. Is cytotoxic chemotherapy recommended as a first-line treatment in patients with PS 0–1 and younger than 75 years old, when their tumor is driver oncogene-negative and PD-L1 is < 50%, or unknown?
CQ 21. Is cytotoxic chemotherapy recommended as a first-line treatment in patients with PS 0–1 and older than 75 years old, when their tumor is driver oncogene-negative and PD-L1 is < 50%, or unknown?
CQ 22. Is cytotoxic chemotherapy recommended as a first-line treatment in patients with PS 2, when their tumor is driver oncogene-negative and PD-L1 is < 50%, or unknown?
CQ 23. Is addition of PD-1/PD-L1 inhibitor to platinum- containing chemotherapy recommended in patients with PS 0–2, when their tumor is driver oncogene-negative and PD-L1 is < 50%, or unknown?
CQ 24. What is the recommended number of courses of platinum-containing chemotherapy?
CQ 25. Is addition of bevacizumab to platinum-doublet chemotherapy recommended in patients with PS 0–2?
CQ 26. Is maintenance therapy recommended in patients who received four courses of platinum-containing chemotherapy without disease progression and with tolerable toxicity?
CQ 27. Is chemotherapy recommended in patients with PS 3–4, when their tumor is driver oncogene-negative or unknown?
**Second- or further-line treatment in patients who are driver oncogene-negative and PD-L1 is < 50 unknown**
CQ 28. Is chemotherapy recommended as a second- or further-line treatment in patients with PS 0–2 who progressed with first-line treatment other than PD-1/PD-L1 inhibitor?
CQ 29. What is the recommended chemotherapy as the second- or further-line treatment in patients with PS 0–2?
CQ 30. Is addition of RAM to DTX recommended in the second-line treatment?
CQ 31. Is erlotinib recommended in the second-line treatment?

### Driver oncogene-positive

#### Treatment of driver oncogene-positive patients (non-squamous cell lung cancer) (Fig. 3)

**Fig. 3:**
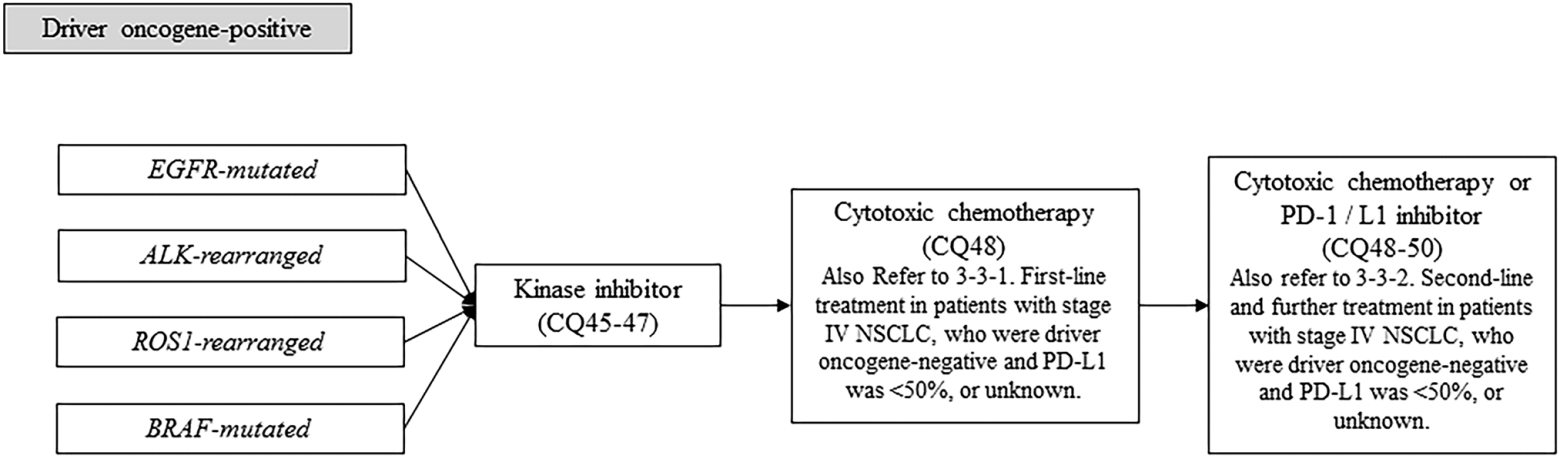
Treatment strategy in driver oncogene-positive NSCLC, stage IV. *EGFR* epidermal growth factor receptor, *ALK* anaplastic lymphoma kinase, *PD-1* programed cell death-1, *PD-L1* programed death-ligand 1, *NSCLC* non-small cell lung cancer

##### CQ 1

What is the optimal first-line treatment for patients who have driver oncogene with good PS (0–1)?

Recommendation:

Kinase inhibitors targeting each oncogene are strongly recommended for patients who have driver oncogene with good PS (0–1).

Recommendation: 1

Evidence level: A

Agreement rate 100%

Comments:

In this guideline, we generically name *EGFR mutation, ALK translocation, ROS1 translocation, and BRAF mutation* as driver oncogenes that might be the direct cause of cancer evolution. Among those patients who harbor with such oncogenic drivers and have good PS, these inhibitors demonstrated both ORR and PFS improvement. Phase III trials were conducted in *EGFR-* and *ALK-mutated* patients, and kinase inhibitors have been shown to be more effective compared with cytotoxic chemotherapy [6–10, 12, 26]. For other types of genetic alterations, phase III trials have not been conducted due to their rarity [i.e., *EGFR uncommon mutation* (see CQ 10), *ROS1 translocation* (see CQ 15), *and BRAF mutation* (see CQ 16)]. Instead, similar efficacy results were observed in phase II trials or subset analyses of phase III trials (i.e., afatinib in *EGFR uncommon mutation*, crizotinib in *ROS1 mutation*) [13–16, 27]. A prospective observational study in the US analyzed 10 genes in 733 patients, and oncogenic drivers were detected among 466 patients (64%). The study also showed that the patients who had oncogenic drivers and received kinase inhibitors lived longer than those who had oncogenic drivers, but did not receive inhibitors (3.5 years versus 2.4 years, propensity score-adjusted hazard ratio: 0.69, 95% CI: 0.53–0.90, *p* = 0.006) [25]. As there are limited data regarding ICIs in patients with oncogenic drivers, current data do not show superiority of ICIs to efficacy of TKIs (see CQ 7).

OS was not improved in phase III trials that compared TKIs with cytotoxic chemotherapy, because most of the patients in cytotoxic chemotherapy arm received kinase inhibitors after progression. A large observational study in *EGFR-mutated* patients demonstrated that PFS with erlotinib did not differ, regardless of treatment line [24]. A firm conclusion regarding the treatment sequence between EGFR-TKI and cytotoxic chemotherapy cannot be drawn. Molecular-targeted drugs usually showed milder toxicity than cytotoxic chemotherapy, but AE profiles and severity are divers.

Based on this evidence, kinase inhibitors targeting each oncogene are recommended treatment for patients who have driver oncogene with good PS (0–1). We judged quality of evidence as A and strength of recommendation as 1. The results of voting by committee members to determine this recommendation are described below.

Involved members regarding this voting: sub-committee on chemotherapy (medical doctors, pharmacists, nurses, statisticians, and patients)

**Table:** 

Benefit with strong recommendation	Benefit with weak recommendation	Unable to determine recommendation	No benefit or risk with weak recommendation	No benefit or risk with strong recommendation
100% (27/27)	0	0	0	0

##### CQ 2

What is the optimal first-line treatment for patients who have driver oncogene with poor PS (2–4)?

Recommendation:

- Kinase inhibitors targeting each oncogene are strongly recommended for patients who have driver oncogene with PS of 2.
- Kinase inhibitors targeting each oncogene are weakly recommended for patients who have driver oncogene with PS of 3–4.

Comments:

- In the phase III trials in *EGFR-mutated* or ALK-rearranged patients, about 5–10% of the participants had PS of 2. Their efficacy was relatively similar to those with PS of 0–1 [8, 9, 12]. Regarding gefitinib and alectinib, prospective trials were reported in *EGFR-mutated* or *ALK-rearranged* patients with poor PS [17, 18].
  Although data are limited in patients who have other types of driver oncogenes, based on the results of patients with *EGFR* or *ALK alteration*, we strongly recommend administration of kinase inhibitors in this population. We judged quality of evidence as C and strength of recommendation as 1. The results of voting by committee members to determine this recommendation are described below.

Involved members regarding this voting: sub-committee on chemotherapy (medical doctors, pharmacists, nurses, statisticians, and patients)

**Table:** 

Benefit with strong recommendation	Benefit with weak recommendation	Unable to determine recommendation	No benefit or risk with weak recommendation	No benefit or risk with strong recommendation
96% (27/28)	4% (1/28)	0	0	0

(b) Gefitinib or alectinib showed beneficial results in the two prospective trials of *EGFR-* or *ALK-altered* patients with poor PS. The number of patients with PS 3–4 was small (22 and 6, relatively), but there were no serious safety issues [17, 18]. In the gefitinib trial, ORR was 66%, and 79% of the patients experienced PS improvement. For other types of genetic alterations (i.e., *EGFR uncommon mutation, ROS1 translocation*, and *BRAF mutation*), data of the patients with PS 3–4 are very limited, but efficacy may be expected in patients with PS 2.
  On the other hand, AE profiles and severity are divers among the drugs. Some of the agents require higher frequencies of drug discontinuation or decrease, even in patients with PS 0–1, and more caution should be paid in case of patients with PS 3–4.

We judged quality of evidence as C, and strength of recommendation as 2. The results of voting by committee members to determine this recommendation are described below.

**Table:** 

Benefit with strong recommendation	Benefit with weak recommendation	Unable to determine recommendation	No benefit or risk with weak recommendation	No benefit or risk with strong recommendation
7% (2/28)	93% (26/28)	0	0	0

##### CQ 3

What is the optimal first-line treatment for elderly (≥ 75 years) patients who have driver oncogene?

Recommendation:

Kinase inhibitors targeting each oncogene are strongly recommended for patients who are elderly (≥ 75 years).

Recommendation: 1

Evidence level: C

Agreement rate 96%

Comments:

In a phase II trial in elderly (≥ 75 years) patients with *EGFR mutation*, gefitinib demonstrated comparable efficacy (ORR of 74% and mPFS of 12.3 months) and safety compared with prior trials that included younger patients [28]. Erlotinib demonstrated similar efficacy regardless of age in a Japanese phase II trial [29].

No data are available regarding elderly (≥ 75 years) patients with other genetic alterations (i.e., *EGFR uncommon mutation, ROS1 translocation, BRAF mutation*), and kinase inhibitors usually showed milder toxicity than cytotoxic chemotherapy. Thus, administration of kinase inhibitors in the elderly is relatively feasible. Based on the results of EGFR-TKIs in the elderly, we recommend the use of any kinase inhibitor for elderly patients who have driver oncogene. We judged quality of evidence as C and strength of recommendation as 1. Note that more attention to adverse events should be paid in this population than in younger patients.

The results of voting by committee members to determine this recommendation are described below.

Involved members regarding this voting: sub-committee on chemotherapy (medical doctors, pharmacists, nurses, statisticians, and patients)

**Table:** 

Benefit with strong recommendation	Benefit with weak recommendation	Unable to determine recommendation	No benefit or risk with weak recommendation	No benefit or risk with strong recommendation
96% (27/28)	4% (1/28)	0	0	0

##### CQ 4

Is cytotoxic chemotherapy recommended for patients who have driver oncogene?

Recommendation:

Cytotoxic chemotherapy is strongly recommended even in patients who have driver oncogene.

Recommendation: 1

Evidence level: A

Agreement rate 100%.

Comments:

Kinase inhibitors are the key drugs in patients with driver oncogenes. However, most of the patients received cytotoxic chemotherapy before or after kinase inhibitors in the phase III trials. Post hoc analyses represented that those patients who received cytotoxic chemotherapy had better prognosis compared with those who did not in prospective trials [30, 31], and similar tendency was shown in a large-scale Japanese observational study [32]. Among *EGFR-mutated* patients, efficacy of chemotherapy may not be different from that in *EGFR wild-type* patients, although no prospective trial has been conducted. Thus, previous evidence, including mixed types of *EGFR mutation*, can apply to this CQ (also see CQ 5, 20, 21 and 23).

Based on this evidence, we strongly recommend the use of cytotoxic chemotherapy even in patients who have driver oncogene during their lines of treatment. We judged quality of evidence as A and strength of recommendation as 1. The results of voting by committee members to determine this recommendation are described below.

Involved members regarding this voting: sub-committee on chemotherapy (medical doctors, pharmacists, nurses, statisticians, and patients)

**Table:** 

Benefit with strong recommendation	Benefit with weak recommendation	Unable to determine recommendation	No benefit or risk with weak recommendation	No benefit or risk with strong recommendation
100% (28/28)	0	0	0	0

##### CQ 5

Is combination of cytotoxic chemotherapy and ICI recommended for patients who have driver oncogene?

Recommendation:

There is no clear evidence to recommend the combination of cytotoxic chemotherapy and ICI in patients who have driver oncogene.

Unable to determine recommendation

Comments:

In the first-line setting, a phase III trial (IMpower150) was conducted to compare carboplatin (CBDCA) + paclitaxel (PTX) + bevacizumab with or without atezolizumab in non-squamous, non-small cell lung cancer. In the subgroup analysis of *EGFR- or ALK-altered* population, PFS was significantly prolonged (median 9.7 months versus 6.1 months (HR 0.89, 95%CI: 0.37–0.94) in atezolizumab arm, while, in interim analysis, OS was not prolonged (median not reached versus 17.5 months (HR 0.54, 95%CI: 0.29–1.03) [21, 33]. However, this result should be interpreted with caution, because this was not a pre-planned analysis and mutation status was not defined as an allocation factor. Atezolizumab arm showed more AEs (≥ grade 3) than control arm (58.5% vs. 50.0%), including immune-related AEs such as diarrhea (20.6% vs. 15.2%) and skin rash (13.3% vs. 5.1%). The guideline committee determined that there is insufficient evidence to draw a firm conclusion based on this subset analysis at this time.

The results of voting by committee members to determine this recommendation are described below.

Involved members regarding this voting: sub-committee on chemotherapy (medical doctors, pharmacists, nurses, statisticians, and patients)

**Table:** 

Benefit with strong recommendation	Benefit with weak recommendation	Unable to determine recommendation	No benefit or risk with weak recommendation	No benefit or risk with strong recommendation
0%	30% (7/23)	65% (15/23)	0	4% (1/23)

##### CQ 6

Is ICI recommended for patients who have driver oncogene?

Recommendation:

There is no clear evidence to recommend ICI in patients who have driver oncogene.

Unable to determine recommendation

Comments:

In a phase III trial (KEYNOTE-024) comparing pembrolizumab with platinum-doublet chemotherapy in chemo-naïve patients whose tumor was positive for PD-L1 ≥ 50%, patients who had *EGFR or ALK alterations* were excluded. In a pooled analysis of phase III trials that compared ICIs (nivolumab, pembrolizumab, and atezolizumab) with docetaxel (DTX), HR of OS comparing ICIs with DTX was 1.05 (95%CI: 0.70–1.55, p < 0.81) [34]. In a single-center analysis, ORR of ICIs was only 3.8% in patients who were positive for *EGFR or ALK* [35]. This evidence may suggest that ICI does not function similarly in patients with driver oncogene as in those without. However, the studies contained relatively small numbers of subjects. The guideline committee finally determined that there is insufficient evidence to draw a firm conclusion on this CQ.

The results of voting by committee members to determine this recommendation are described below.

Involved members regarding this voting: sub-committee on chemotherapy (medical doctors, pharmacists, nurses, statisticians, and patients)

**Table:** 

Benefit with strong recommendation	Benefit with weak recommendation	Unable to determine recommendation	No benefit or risk with weak recommendation	No benefit or risk with strong recommendation
0%	23% (5/22)	73% (16/22)	0	5% (1/22)

#### *EGFR-mutated*, non-squamous cell lung cancer

**Fig. 4:**
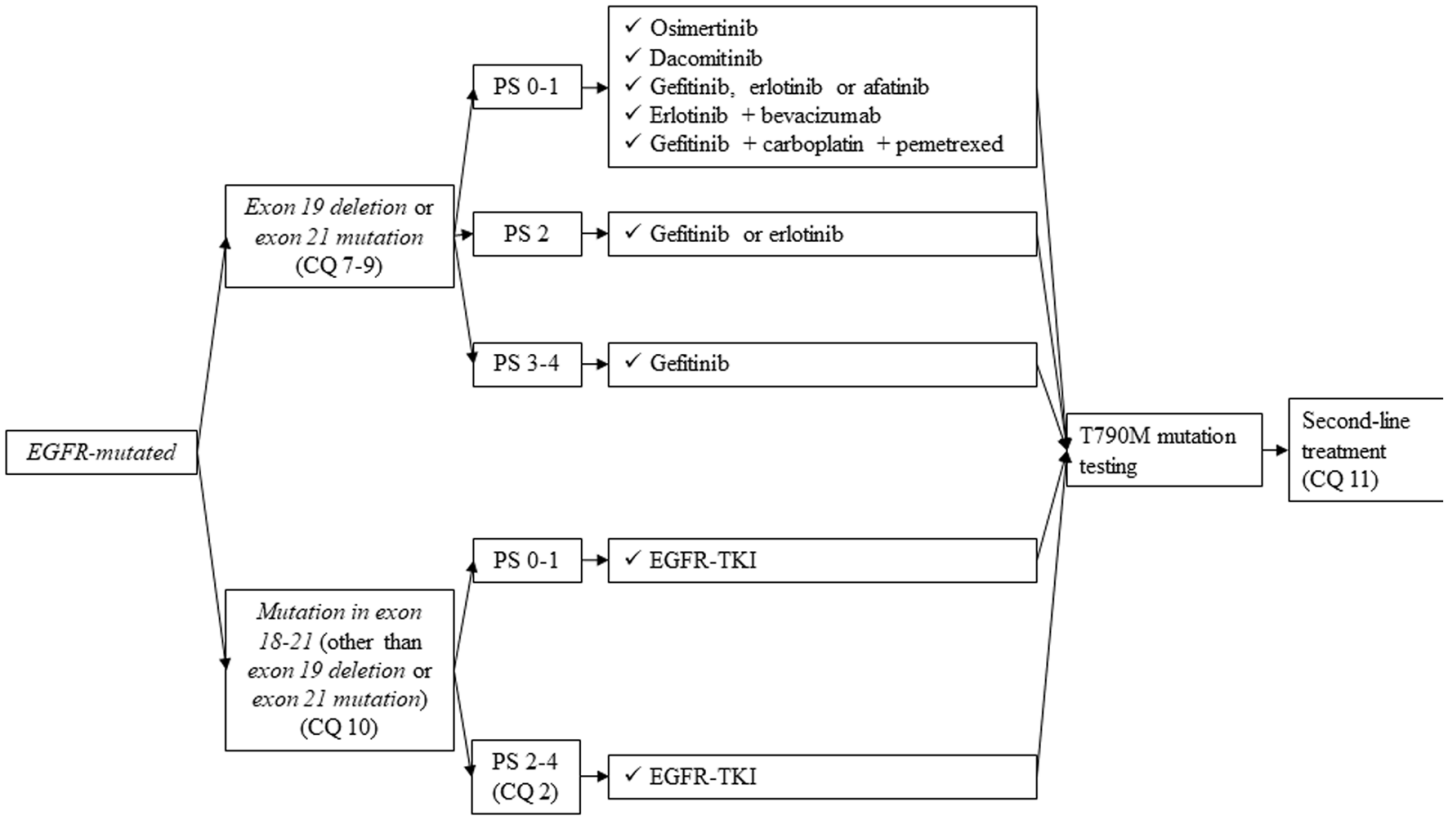
First-line treatment of *EGFR-mutated* NSCLC, stage IV. *EGFR* epidermal growth factor receptor, *TKI* tyrosine-kinase inhibitor, *PS* performance status

*First-line treatment in patients who have EGFR mutation* (*exon 19 deletion or L858R mutation) is shown in Fig.*4.

##### CQ 7

What is the recommended first-line treatment in patients who have *EGFR mutation* (*exon 19 deletion* or *L858R mutation*) with PS of 0–1?

Recommendation:

- Osimertinib is strongly recommended:

Recommendation: 1

Evidence level: B

Agreement rate 83%

(b) Dacomitinib is weakly recommended:

Recommendation: 2

Evidence level: B

Agreement rate 83%

(c) Gefitinib, erlotinib, or afatinib are weakly recommended:

Recommendation: 2

Evidence level: A

Agreement rate 100%

(d) Erlotinib plus bevacizumab is weakly recommended:

Recommendation: 2

Evidence level: B

Agreement rate 92%

(e) Gefitinib combined with carboplatin plus pemetrexed is weakly recommended:

Recommendation: 2

Evidence level: B

Agreement rate 92%

Comments:

*EGFR Exon 19 deletion* and *L858R mutation* consist of about 90% of *EGFR mutation* and are known as sensitive to EGFR-TKIs. In phase III trials comparing EGFR-TKI (gefitinib, erlotinib, or afatinib) with platinum-doublet chemotherapy, types of *EGFR mutation* were limited to or consisted mostly of these two mutations [6–10, 26]. All trials demonstrated significant prolongation of PFS in EGFR-TKI arm, and several QOL items were improved [36].

- FLAURA is a phase III trial comparing osimertinib with first-generation EGFR-TKIs (gefitinib or erlotinib) in stage IV NSCLC patients who have *EGFR mutation (exon 19 deletion or L858R mutation*) and PS of 0–1. In this trial, PFS (primary endpoint) was significantly prolonged in osimertinib arm [18.9 months versus 10.2 months, HR 0.46 (95%CI: 0.37–0.57), *p* < 0.001] [37]. Regarding toxicity, first-generation EGFR-TKIs showed diarrhea in 57%, skin rash in 48%, AST elevation in 25%, and interstitial lung disease in 2% of patients, while osimertinib showed milder toxicities (diarrhea 58%, skin rash 25%, AST elevation 9%, and interstitial lung disease 4%).
  Based on these balances between efficacy and toxicity, we strongly recommend osimertinib in patients who have *EGFR mutation* (*exon 19 deletion* or *L858R mutation*) with PS of 0–1. We judged quality of evidence as B and strength of recommendation as 1. The results of voting by committee members to determine this recommendation are described below.

Involved members regarding this voting: sub-committee on chemotherapy (medical doctors, pharmacists, nurses, statisticians, and patients)

**Table:** 

Benefit with strong recommendation	Benefit with weak recommendation	Unable to determine recommendation	No benefit or risk with weak recommendation	No benefit or risk with strong recommendation
83% (19/23)	17% (4/23)	0	0	0

(b) ARCHER1050 was a phase III trial comparing dacomitinib with gefitinib in stage IV NSCLC patients who have *EGFR mutation* (*exon 19 deletion* or *L858R mutation*) and PS of 0–1. In this trial, PFS (primary endpoint) was significantly prolonged in dacomitinib arm [14.7 months versus 9.2 months, HR 0.59 (95%CI: 0.47–0.74), *p* < 0.001]. Regarding secondary endpoint OS, dacomitinib also showed statistically significant prolongation [34.1 months versus 26.8 months, HR 0.76 (95%CI: 0.852–0.993), *p* = 0.044] [38, 39]. However, dacomitinib was more toxic: diarrhea in 78%, paronychia in 54%, and skin rash in 35% of patients, while gefitinib showed diarrhea in 55%, paronychia in 19%, and skin rash in 35%.
  Based on these balances between efficacy and toxicity, we weakly recommend dacomitinib in patients who have *EGFR mutation* (*exon 19 deletion* or *L858R mutation*) with PS of 0–1. We judged quality of evidence as B and strength of recommendation as 2. The results of voting by committee members to determine this recommendation are described below.

Involved members regarding this voting: sub-committee on chemotherapy (medical doctors, pharmacists, nurses, statisticians, and patients)

**Table:** 

Benefit with strong recommendation	Benefit with weak recommendation	Unable to determine recommendation	No benefit or risk with weak recommendation	No benefit or risk with strong recommendation
13% (3/23)	83% (19/23)	0	4% (1/23)	0

(c) No direct comparison showed superiority among first-generation EGFR-TKIs [40].
  In a randomized phase II trial, afatinib demonstrated longer PFS than gefitinib, but toxicity was severe [41].
  Based on these balances between efficacy and toxicity, we weakly recommend gefitinib, erlotinib, or afatinib in patients who have *EGFR mutation* (*exon 19 deletion* or *L858R mutation*) with PS of 0–1. We judged quality of evidence as A and strength of recommendation as 2. The results of voting by committee members to determine this recommendation are described below.

Involved members regarding this voting: sub-committee on chemotherapy (medical doctors, pharmacists, nurses, statisticians, and patients)

**Table:** 

Benefit with strong recommendation	Benefit with weak recommendation	Unable to determine recommendation	No benefit or risk with weak recommendation	No benefit or risk with strong recommendation
0	100% (23/23)	0	0	0

(d) In a Japanese phase III trial, erlotinib plus bevacizumab significantly prolonged PFS compared to erlotinib alone [16.9 months versus 13.3 months, HR 0.605 (95%CI: 0.417–0.877), *p* = 0.01573] [42]. Bevacizumab-related toxicity was observed in combination arm (≥ Gr3 AEs: hypertension 9%, proteinuria 32%, bleeding 26%). In a former phase II trial, PFS was also prolonged [16.0 months versus 9.7 months, HR 0.54 (95%CI: 0.36–0.79)], but there was no significant difference in OS [43, 44].
  Based on this evidence, we weakly recommend erlotinib plus bevacizumab in patients who have *EGFR mutation* (*exon 19 deletion* or *L858R mutation*) with PS of 0–1. We judged quality of evidence as B and strength of recommendation as 2. The results of voting by committee members to determine this recommendation are described below.

Involved members regarding this voting: sub-committee on chemotherapy (medical doctors, pharmacists, nurses, statisticians, and patients)

**Table:** 

Benefit with strong recommendation	Benefit with weak recommendation	Unable to determine recommendation	No benefit or risk with weak recommendation	No benefit or risk with strong recommendation
4% (1/23)	92% (21/23)	0	4% (1/23)	0

(e) In a phase III trial, gefitinib combined with carboplatin plus pemetrexed (PEM) was compared with gefitinib monotherapy. PFS (one of the primary endpoints) was significantly prolonged in combination arm [20.9 months versus 11.2 months, HR 0.484 (95%CI: 0.391–0.625), *p* < 0.01], but there was no difference in PFS2* [20.9 months versus 20.7 months, HR 0.966 (95%CI: 0.766–1.220), *p* = 0.774]. Combination arm showed OS of 52.2 months, while 38.8 months was shown in monotherapy arm (HR 0.695). Hematological toxicity (≥ Gr3) was frequently observed in the combination arm [45].
  Based on this evidence, we weakly recommend erlotinib plus bevacizumab in patients who have *EGFR mutation* (*exon 19 deletion* or *L858R mutation*) with PS of 0–1. We judged quality of evidence as B and strength of recommendation as 2. The results of voting by committee members to determine this recommendation are described below.

*PFS2 in monotherapy arm consisted of PFS with gefitinib plus that with subsequent therapy, while PFS2 in combination arm consisted of PFS with gefitinib combined with carboplatin plus pemetrexed.

Involved members regarding this voting: sub-committee on chemotherapy (medical doctors, pharmacists, nurses, statisticians, and patients)

**Table:** 

Benefit with strong recommendation	Benefit with weak recommendation	Unable to determine recommendation	No benefit or risk with weak recommendation	No benefit or risk with strong recommendation
0	92% (21/23)	4% (1/23)	4% (1/23)	0

##### CQ 8

What is the recommended first-line treatment in patients who have *EGFR mutation* (*exon 19 deletion* or *L858R mutation*) with PS of 2?

Recommendation:

Gefitinib or erlotinib is strongly recommended.

Recommendation: 1

Evidence level: C

Agreement rate 100%

Comments:

In the two phase III trials in metastatic NSCLC patients with *EGFR mutation* comparing erlotinib with platinum-doublet chemotherapy, 7% and 14% of the patients had PS of 2 [8, 9]. The efficacy in those patients was relatively similar to that in those with PS of 0–1. Gefitinib showed efficacy in prospective trials in patients with poor PS [28, 46]. Data are insufficient regarding efficacy and safety of afatinib and dacomitinib in this population [10, 26, 38]. Although the situation is similar, osimertinib can be considered, because the toxicity is generally milder than that of gefitinib or erlotinib, except for interstitial lung disease [37].

Based on this evidence, we strongly recommend the administration of gefitinib or erlotinib in patients who have *EGFR mutation* (*exon 19 deletion* or *L858R mutation*) with PS of 2. We judged quality of evidence as C and strength of recommendation as 1. The results of voting by committee members to determine this recommendation are described below.

The results of voting by committee members to determine this recommendation are described below.

Involved members regarding this voting: sub-committee on chemotherapy (medical doctors, pharmacists, nurses, statisticians, and patients)

**Table:** 

Benefit with strong recommendation	Benefit with weak recommendation	Unable to determine recommendation	No benefit or risk with weak recommendation	No benefit or risk with strong recommendation
100% (24/24)	0	0	0	0

##### CQ 9

What is the recommended first-line treatment in patients who have *EGFR mutation* (*exon 19 deletion* or *L858R mutation*) with PS of 3–4?

Recommendation:

Gefitinib is strongly recommended.

Recommendation: 1

Evidence level: C

Agreement rate 75%

Comments:

In a prospective trial of gefitinib in *EGFR-mutated* patients with poor PS (mostly PS 3–4), PS was improved among about 80% of the patients and efficacy was good (ORR 66%, median PFS 6.5 months and median OS 17.8 months) [46]. On the other hand, administration should be considered carefully, because poor PS is a well-known risk factor of interstitial lung disease, as are male, smoking history, pre-existing interstitial lung disease, patients who have limited normal lung region, and those with heart disease [40, 41]. The guideline committee had thorough discussion regarding patients with PS of 4. For such patients, physicians must consider whether EGFR-TKI would improve PS, or symptoms that are more meaningful outcomes than good PS.

Based on this evidence, we strongly recommend the administration of gefitinib in patients who have *EGFR mutation* (*exon 19 deletion* or *L858R mutation*) with PS of 3–4. We judged quality of evidence as C and strength of recommendation as 1. The results of voting by committee members to determine this recommendation are described below.

Involved members regarding this voting: sub-committee on chemotherapy (medical doctors, pharmacists, nurses, statisticians, and patients)

**Table:** 

Benefit with strong recommendation	Benefit with weak recommendation	Unable to determine recommendation	No benefit or risk with weak recommendation	No benefit or risk with strong recommendation
75% (18/24)	25% (6/24)	0	0	0

*First-line treatment of those patients who have EGFR mutation other than exon 19 deletion or L858R mutation*

##### CQ 10

What is the recommended first-line treatment in patients who have *EGFR mutation* other than *exon 19 deletion or L858R mutation*, with PS of 0–1?

Recommendation:

- Gefitinib, erlotinib, or afatinib is weakly recommended in patients who have *EGFR mutation of exon18-21*, except for *exon 19 deletion, L858R mutation, exon20 insertion*, and *T790M mutation*, with PS of 0–1.

Recommendation: 2

Evidence level: C

Agreement rate 87%

(b) EGFR-TKI is not strongly recommended in patients who have *EGFR exon 20 insertion mutation* with PS of 0–1.

Recommendation: 1

Evidence level: C

Agreement rate 70%

(c) Osimertinib is weakly recommended in patients who have de novo *EGFR exon 20 T790M mutation* with PS of 0–1.

Recommendation: 2

Evidence level: D

Agreement rate 67%

Comments:

- Among *EGFR mutations, exon19 deletion* and *exon 21 L858R mutation* comprise about 90% [49]

Other mutations are called “*uncommon mutations*,” and are located between exon 18 and 21 (i.e., *E709X, G719X, S768I, P848L, L861Q, or exon20 insertion)*. They are usually sensitive to EGFR-TKIs, but ORR was slightly lower than that observed in common mutation [50]. Most of the phase III trials completely excluded these mutations [6, 8, 9], or the mutations existed in only 10% of the entire population [7, 10, 26].

Among patients with *uncommon mutation* other than *exon20 insertion* and *T790M mutation*, ORR was reported as 48–71% [16, 50]. Of those, ORR with afatinib in a small subset of a trial was 71.1%, which showed relatively higher tendency than other EGFR-TKIs [16]. Although this result was obtained in a prospective manner, a firm conclusion as to recommend afatinib as better than other EGFR-TKIs cannot be drawn.

Based on this evidence, gefitinib, erlotinib, or afatinib is weakly recommended in patients who have *EGFR mutation* of *exon18-21*, except for *exon 19 deletion, L858R mutation, exon20 insertion*, and *T790M mutation*, with PS of 0–1. We judged quality of evidence as C and strength of recommendation as 2. The results of voting by committee members to determine this recommendation are described below.

Involved members regarding this voting: sub-committee on chemotherapy (medical doctors, pharmacists, nurses, statisticians, and patients)

**Table:** 

Benefit with strong recommendation	Benefit with weak recommendation	Unable to determine recommendation	No benefit or risk with weak recommendation	No benefit or risk with strong recommendation
13% (3/24)	87% (21/24)	0	0	0

(b) There are few data regarding *EGFR exon 20 insertion*, and ORR in these reports was around 10% [16, 51]. Thus, EGFR-TKI was not considered as a first-line treatment option.

Based on this evidence, EGFR-TKI is not strongly recommended in patients who have *EGFR exon 20 insertion mutation* with PS of 0–1. We judged quality of evidence as C and strength of recommendation as 1. The results of voting by committee members to determine this recommendation are described below.

Involved members regarding this voting: sub-committee on chemotherapy (medical doctors, pharmacists, nurses, statisticians, and patients)

**Table:** 

Benefit with strong recommendation	Benefit with weak recommendation	Unable to determine recommendation	No benefit or risk with weak recommendation	No benefit or risk with strong recommendation
0%	4% (1/27)	0	26% (7/27)	70% (19/27)

(c) There are few data regarding *de novo EGFR exon 20 T790M mutation*. In the FLAURA study, only 5 of 556 patients had *de novo* T790M mutation. On the other hand, six of seven de novo T790M mutation patients experienced PR in the phase I trial of osimertinib [52]. In patients with *EGFR T790M mutation* after progression of EGFR-TKIs, osimertinib showed good efficacy results in the phase III trial. This indirectly suggests that similar efficacy may be expected in patients with *de novo* T790M mutation.
  Based on this evidence, osimertinib is weakly recommended in patients who have *de novo EGFR exon 20 T790M mutation* with PS of 0–1. We judged quality of evidence as D and strength of recommendation as 2. The results of voting by committee members to determine this recommendation are described below.

Involved members regarding this voting: sub-committee on chemotherapy (medical doctors, pharmacists, nurses, statisticians, and patients)

**Table:** 

Benefit with strong recommendation	Benefit with weak recommendation	Unable to determine recommendation	No benefit or risk with weak recommendation	No benefit or risk with strong recommendation
4% (1/27)	67% (18/27)	29% (8/27)	0	0

**Fig. 5:**
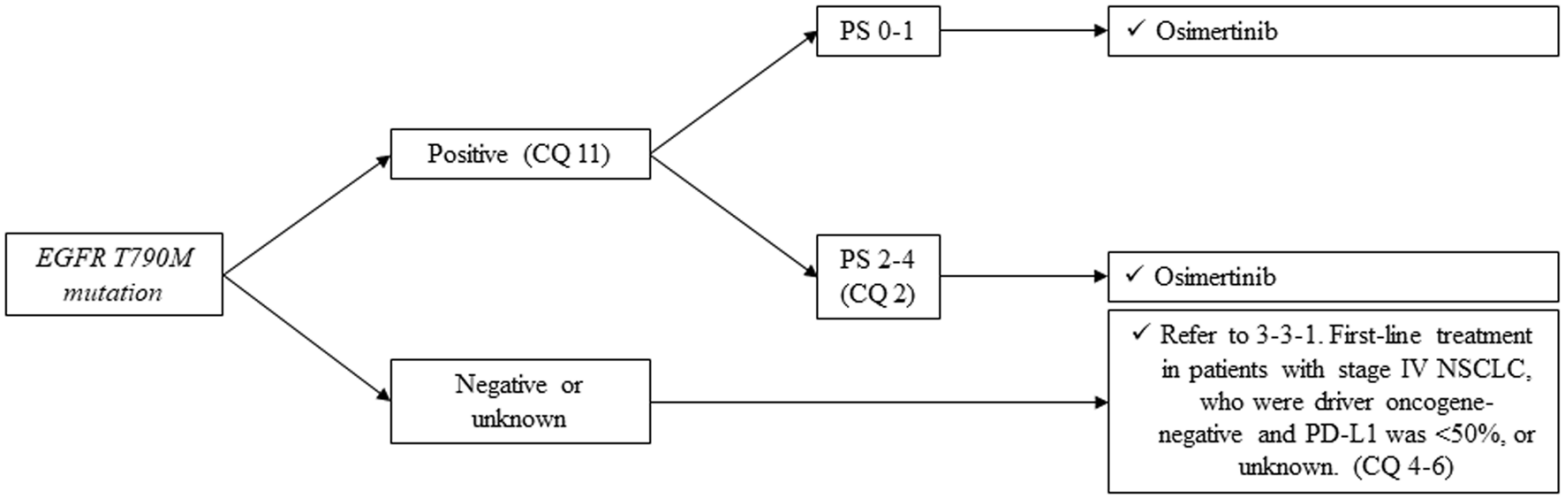
Second-line or further treatment of *EGFR-mutated* NSCLC, stage IV. *EGFR* epidermal growth factor receptor, *ALK* anaplastic lymphoma kinase, *TKI* tyrosine-kinase inhibitor, *PS* performance status, *NSCLC* non-small cell lung cancer

*Second-line and further treatment of those patients who have EGFR mutation (Fig. *5*)*.

##### CQ 11

What is the recommended second-line treatment in patients *with EGFR T790M mutation* after progression of EGFR-TKIs?

Recommendation:

Osimertinib is strongly recommended.

Recommendation: 1

Evidence level: B

Agreement rate 100%

Comments:

Osimertinib is a third-generation EGFR-TKI that has a potent activity to both sensitive *EGFR mutation* and resistant *T790M mutation*. A phase III trial comparing cytotoxic chemotherapy with osimertinib was conducted in those patients who had *EGFR T790M mutation* after progression of the first- or second-generation EGFR-TKIs (gefitinib, erlotinib, or afatinib) [53]. PFS (primary endpoint) was significantly prolonged in osimertinib arm [10.1 months versus 4.4 months, HR 0.30 (95%CI: 0.23-0.41), *p* < 0.001]. There were fewer adverse events greater than grade 3 in osimertinib arm (6% versus 34%).

Based on this evidence, we strongly recommend the administration of osimertinib in patients *with EGFR T790M mutation* after progression of EGFR-TKIs. We judged quality of evidence as B and strength of recommendation as 1. The results of voting by committee members to determine this recommendation are described below.

The results of voting by committee members to determine this recommendation are described below.

Involved members regarding this voting: sub-committee on chemotherapy (medical doctors, pharmacists, nurses, statisticians, and patients)

**Table:** 

Benefit with strong recommendation	Benefit with weak recommendation	Unable to determine recommendation	No benefit or risk with weak recommendation	No benefit or risk with strong recommendation
100% (26/26)	0	0	0	0

#### *ALK-rearranged*, non-squamous cell lung cancer

**Fig. 6:**
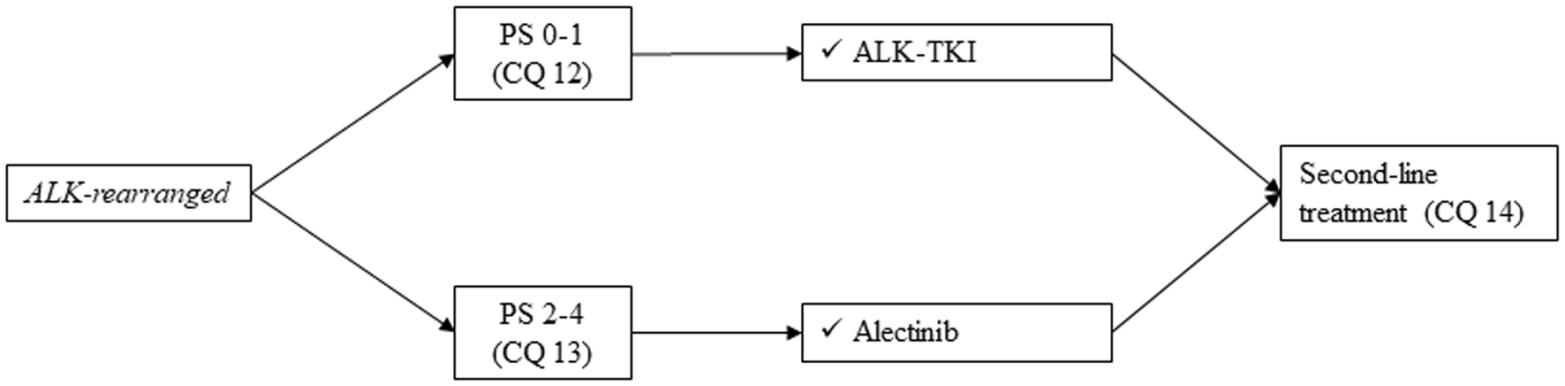
First-line treatment of *ALK-rearranged* NSCLC, stage IV. Abbreviations: ALK; anaplastic lymphoma kinase, TKI; tyrosine-kinase inhibitor, PS; performance status

*First-line treatment of those patients who have ALK rearrangement is shown in Fig. *6.

##### CQ 12

What is the recommended first-line treatment in patients who have *ALK rearrangement* with PS of 0–1?

Recommendation:

- Alectinib is strongly recommended.

Recommendation: 1

Evidence level: A

Agreement rate 100%

(b) Crizotinib is weakly recommended.

Recommendation: 2

Evidence level: A

Agreement rate 100%

(c) Ceritinib is weakly recommended.

Recommendation: 2

Evidence level: B

Agreement rate 100%

Comments:

In Japan, alectinib, crizotinib, and ceritinib can be used as the first-line treatment for advanced SCLC patients who have *ALK rearrangement*. Regarding crizotinib and ceritinib, these were compared with platinum-doublet chemotherapy in phase III trials. Crizotinib showed significant prolongation of PFS [10.9 months versus 7.0 months, HR 0.45 (95% CI: 0.35–0.60), *p* < 0.0001] and improvement in ORR (74% versus 45%) [54]. Ceritinib showed significant prolongation of PFS [16.6 months versus 8.1 months, HR 0.55 (95% CI: 0.42–0.73), *p* < 0.0001] and improvement in ORR (72.5% versus 26.7%) [55].

Alectinib was compared with crizotinib in phase III trials and showed significant prolongation of PFS [25.9 months versus 10.2 months, HR 0.38 (95% CI: 0.26–0.55), *p* < 0.0001] in a Japanese trial. Similar results were also replicated in a US trial [HR 0.47 (95% CI: 0.34–0.65), *p* < 0.001], although 600 mg of alectinib was administered [56–58]. Treatment-related adverse events were observed in 52% of patients in crizotinib arm, while 26% in alectinib arm [56].

Based on the current data, we strongly recommend the administration of alectinib in patients who have *ALK rearrangement* with PS of 0–1. We judged quality of evidence as A and strength of recommendation as 1. On the other hand, OS data in these trials were immature. The superiority of alectinib in terms of OS is unknown.

In a phase I/II trial and phase II trials, crizotinib followed by alectinib showed relatively good efficacy (ORR of 48–50% and median PFS of 8.1–8.9 months) [59–61]. Thus, crizotinib can be an alternative first-line treatment. We judged quality of evidence as A and strength of recommendation as 2.

Similarly, ceritinib can be considered as an alternative, but no comparative study with other ALK-TKI has been conducted. Treatment-related adverse events were seen in 65% of the patients, and liver dysfunction was the main event [55]. We judged quality of evidence as B and strength of recommendation as 2.

Regarding elderly patients, 16% of the participants were ≥ 65 years old in the phase III trial comparing crizotinib with platinum-doublet chemotherapy, and efficacy was similar between elderly and younger patients [54]. In addition, 11% of the participants were ≥ 75 years old in the phase III trial comparing alectinib with crizotinib, and advantage of alectinib over crizotinib was similar among the elderly.

The results of voting by committee members to determine this recommendation are described below.

Involved members regarding this voting: sub-committee on chemotherapy (medical doctors, pharmacists, nurses, statisticians, and patients)

(a)

**Table:** 

Benefit with strong recommendation	Benefit with weak recommendation	Unable to determine recommendation	No benefit or risk with weak recommendation	No benefit or risk with strong recommendation
100% (27/27)	0	0	0	0

(b)

**Table:** 

Benefit with strong recommendation	Benefit with weak recommendation	Unable to determine recommendation	No benefit or risk with weak recommendation	No benefit or risk with strong recommendation
0	96% (26/27)	0	0	4% (1/27)

(c)

**Table:** 

Benefit with strong recommendation	Benefit with weak recommendation	Unable to determine recommendation	No benefit or risk with weak recommendation	No benefit or risk with strong recommendation
0	96% (26/27)	0	0	4% (1/27)

##### CQ 13

What is the recommended first-line treatment in patients who have *ALK rearrangement* with PS of 2–4?

Recommendation:

Alectinib is strongly recommended.

Recommendation: 1

Evidence level: C

Agreement rate 100%

Comments:

One study reported the efficacy of alectinib in patients who have *ALK rearrangement* with poor PS [18]. Although the number of patients was relatively small (PS 2: 12 patients, PS 3: 5 patients, and PS 4: 1 patient), there was no severe adverse event. In this trial, ORR was 72%. In the phase III trial comparing alectinib with crizotinib, there were fewer adverse events in the alectinib arm, although only 2% of the patients had PS of 2 [56].

Based on this evidence, we strongly recommend the administration of alectinib in patients who have *ALK rearrangement* with PS of 2–4. We judged quality of evidence as C and strength of recommendation as 1. The results of voting by committee members to determine this recommendation are described below.

Involved members regarding this voting: sub-committee on chemotherapy (medical doctors, pharmacists, nurses, statisticians, and patients)

**Table:** 

Benefit with strong recommendation	Benefit with weak recommendation	Unable to determine recommendation	No benefit or risk with weak recommendation	No benefit or risk with strong recommendation
100% (27/27)	0	0	0	0

**Fig. 7:**
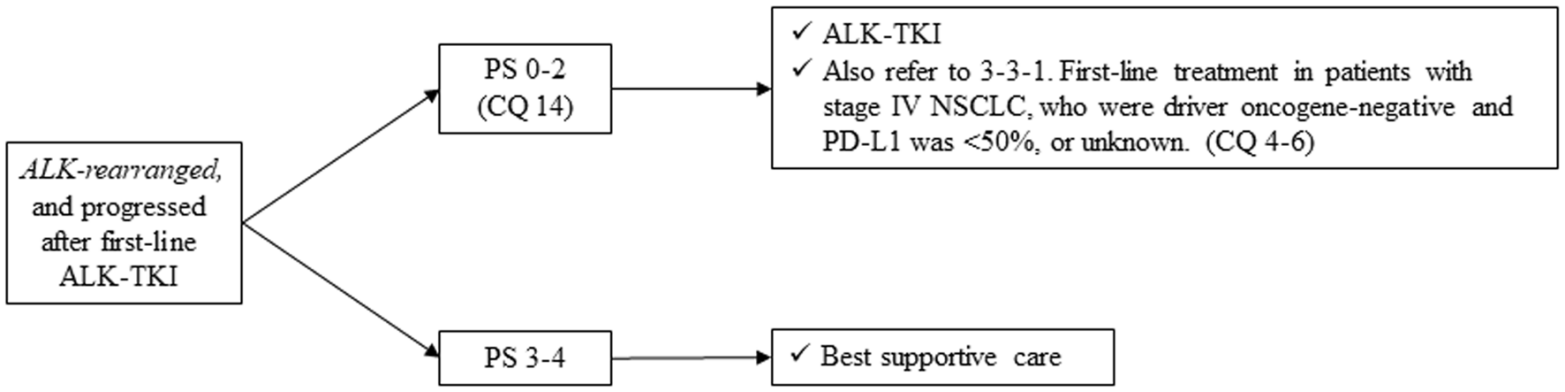
Second-line or further treatment of *ALK-rearranged* NSCLC, stage IV. *PS* performance status, *NSCLC* non-small cell lung cancer

*Second-line treatment of those patients who have ALK rearrangement is shown in Fig. *7.

##### CQ 14

What is the recommended second-line treatment in patients *with ALK rearrangement* after progression of ALK-TKIs?

Recommendation:

- After the first-line treatment of crizotinib, alectinib is strongly recommended:

Recommendation: 1

Evidence level: C

Agreement rate 96%

(b) After the first-line treatment of crizotinib, ceritinib is weakly recommended:

Recommendation: 2

Evidence level: C

Agreement rate 100%

(c) Lorlatinib is weakly recommended:

Recommendation: 2

Evidence level: C

Agreement rate 96%

Comments:

- In a phase I/II trial and phase II trials conducted overseas, crizotinib followed by alectinib in patients with *ALK rearrangement* showed relatively good efficacy (ORR of 48–50% and median PFS of 8.1–8.9 months) [59–61]. In Japan, 23 patients who had progressed with crizotinib were treated with alectinib [62]. In this trial, ORR was 65% and mPFS was 12.9 months. Although there are no comparable data of alectinib with platinum-doublet chemotherapy, these results from single-arm trials can be considered to be at least equivalent.
  Based on this evidence, we strongly recommend the administration of alectinib in crizotinib-refractory, ALK-rearranged patients. We judged quality of evidence as C and strength of recommendation as 1. The results of voting by committee members to determine this recommendation are described below.

Involved members regarding this voting: sub-committee on chemotherapy (medical doctors, pharmacists, nurses, statisticians, and patients)

**Table:** 

Benefit with strong recommendation	Benefit with weak recommendation	Unable to determine recommendation	No benefit or risk with weak recommendation	No benefit or risk with strong recommendation
96% (25/26)	4% (1/26)	0	0	0

(b) In a subgroup analysis of phase I trial of ceritinib in patients with *ALK rearrangement*, 80 patients who were refractory to crizotinib demonstrated ORR of 56% and mPFS of 6.9 months [63]. In a single-arm phase II trial in *ALK-rearranged* patients who progressed with crizotinib and platinum-doublet chemotherapy, ORR was 38.6% and mPFS was 5.7 months. In a phase III study (ASCEND-5) comparing ceritinib with cytotoxic chemotherapy (PEM or DTX), PFS (primary endpoint) was significantly prolonged in ceritinib arm [5.4 months versus 1.6 months, HR 0.49 (95%CI: 0.36–0.67), *p* < 0.001] [65]. In a Japanese phase I trial, 5 of 9 patients who had treatment history with crizotinib had PR with higher GI toxicities [66]. Although there are no comparable data of ceritinib with platinum-doublet chemotherapy, these efficacy results can be considered equivalent, and toxicity may be higher than with alectinib. In addition, the ASCEND-5 study set cytotoxic chemotherapy monotherapy as a control, which makes the results difficult to interpret.

Based on this evidence, we weakly recommend the administration of ceritinib in crizotinib-refractory, *ALK-rearranged* patients. We judged quality of evidence as C and strength of recommendation as 2. The results of voting by committee members to determine this recommendation are described below.

Involved members regarding this voting: sub-committee on chemotherapy (medical doctors, pharmacists, nurses, statisticians, and patients)

**Table:** 

Benefit with strong recommendation	Benefit with weak recommendation	Unable to determine recommendation	No benefit or risk with weak recommendation	No benefit or risk with strong recommendation
0	100% (26/26)	0	0	0

(c) In a phase I study of lorlatinib in patients with *ALK rearrangement*, 41 ALK-TKI pre-treated patients (37 were treated with crizotinib) demonstrated ORR of 46% and mPFS of 9.3 months [66]. In a phase II study of lorlatinib, 59 patients who were refractory to crizotinib showed ORR 72.9% and mPFS of 11.1 months [67]. In this study, 28 patients who progressed with ALK-TKIs other than crizotinib showed ORR of 42.9% and mPFS of 5.5 months.
  Based on this evidence, we weakly recommend the administration of lorlatinib in ALK-rearranged patients who progressed with ALK-TKI. We judged quality of evidence as C and strength of recommendation as 2. The results of voting by committee members to determine this recommendation are described below.

Involved members regarding this voting: sub-committee on chemotherapy (medical doctors, pharmacists, nurses, statisticians, and patients)

**Table:** 

Benefit with strong recommendation	Benefit with weak recommendation	Unable to determine recommendation	No benefit or risk with weak recommendation	No benefit or risk with strong recommendation
4% (1/26)	96% (25/26)	0	0	0

#### *ROS1 rearranged*, non-squamous cell lung cancer (Fig. 8)

**Fig. 8:**
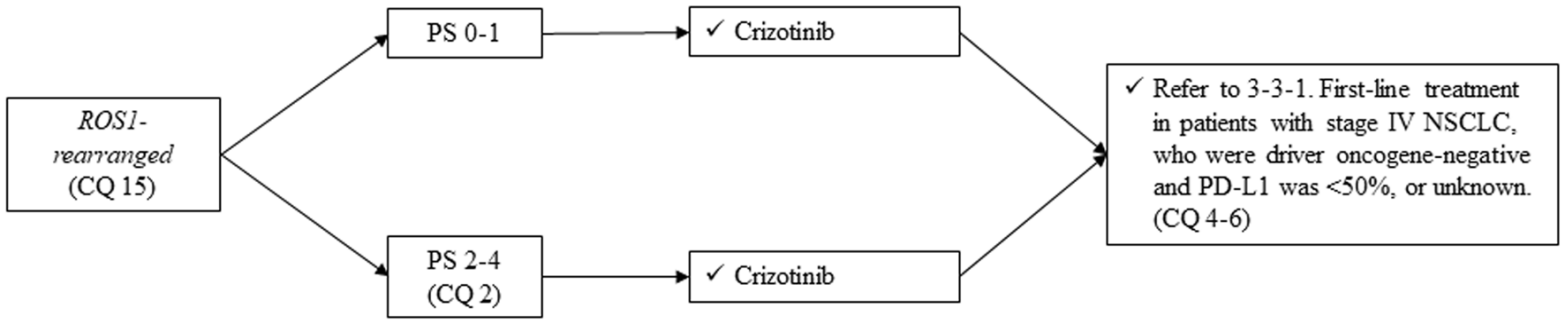
Treatment of *ROS1-rearranged* NSCLC, stage IV. *PS* performance status, *NSCLC* non-small cell lung cancer

##### CQ 15

Is crizotinib a recommended treatment in patients who have *ROS1 rearrangement*?

Recommendation:

Crizotinib is strongly recommended:

Recommendation: 1

Evidence level: C

Agreement rate 100%

Comments:

Several reports mentioned the efficacy of crizotinib in patients who had *ROS1 rearrangement*. In a study conducted mainly in the US, crizotinib demonstrated ORR of 72% and mPFS of 19.2 months in 50 patients [13]. In an East Asian study, 127 patients showed ORR of 69.3% and mPFS of 13.4 months [14]. These results are comparable or better than those for crizotinib in *ALK-rearranged* patients.

Based on this evidence, we strongly recommend the administration of crizotinib in patients who have *ROS1 rearrangement* (also refer to CQ 17). We judged quality of evidence as C and strength of recommendation as 1. The results of voting by committee members to determine this recommendation are described below.

Involved members regarding this voting: sub-committee on chemotherapy (medical doctors, pharmacists, nurses, statisticians, and patients)

**Table:** 

Benefit with strong recommendation	Benefit with weak recommendation	Unable to determine recommendation	No benefit or risk with weak recommendation	No benefit or risk with strong recommendation
100% (26/26)	0	0	0	0

#### *BRAF-mutated*, non-squamous cell lung cancer (Fig. 9)

**Fig. 9:**
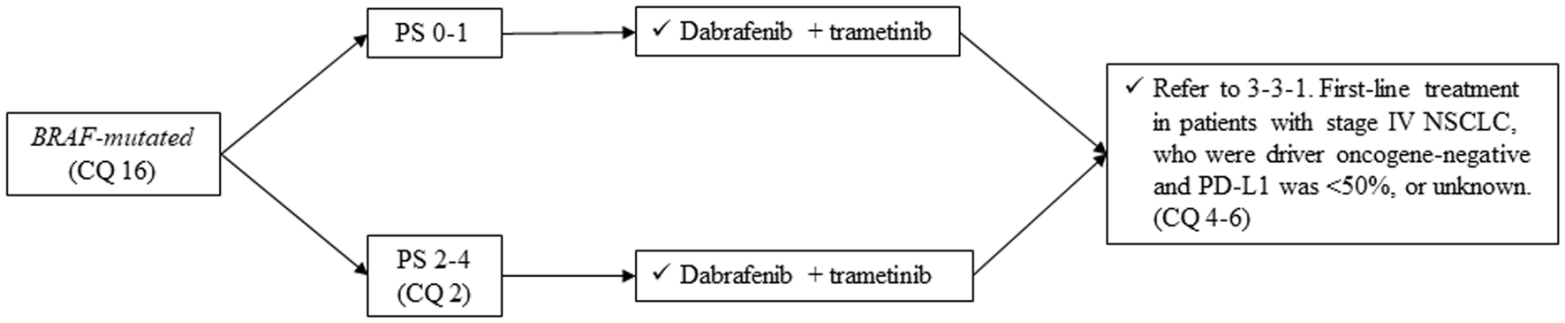
Treatment of *BRAF-mutated* NSCLC, stage IV. *PS* performance status, *NSCLC* non-small cell lung cancer

##### CQ 16

Is dabrafenib plus trametinib a recommended treatment in patients who have *BRAF mutation*?

Recommendation:

Dabrafenib plus trametinib is strongly recommended.

Recommendation: 1

Evidence level: C

Agreement rate 69%

Comments:

In patients with *BRAF mutation*, several reports showed the efficacy of dabrafenib or dabrafenib plus trametinib. In a phase II trial of dabrafenib plus trametinib for *BRAF-mutated* stage IV NSCLC patients who have prior history of chemotherapy, 57 patients showed ORR (primary endpoint) of 66.7% and mPFS of 9.7 months [15]. In another phase II trial, of dabrafenib plus trametinib for *BRAF-mutated* patients who were chemo-naïve, 36 patients showed ORR (primary endpoint) of 64% and mPFS of 10.9 months [27].

Although there were limited numbers of Japanese patients in these trials, we strongly recommend the administration of dabrafenib plus trametinib in patients who have *BRAF mutation* (also refer to CQ 17). We judged quality of evidence as C and strength of recommendation as 1. The results of voting by committee members to determine this recommendation are described below.

Involved members regarding this voting: sub-committee on chemotherapy (medical doctors, pharmacists, nurses, statisticians, and patients)

First voting:

**Table:** 

Benefit with strong recommendation	Benefit with weak recommendation	Unable to determine recommendation	No benefit or risk with weak recommendation	No benefit or risk with strong recommendation
58% (15/26)	42% (11/26)	0	0	0

Second voting:

**Table:** 

Benefit with strong recommendation	Benefit with weak recommendation	Unable to determine recommendation	No benefit or risk with weak recommendation	No benefit or risk with strong recommendation
69% (18/26)	31% (8/26)	0	0	0

#### Driver oncogene-positive, squamous cell lung cancer

##### CQ 17

Is tyrosine-kinase inhibitor a recommended treatment in squamous cell lung cancer patients who have driver oncogene?

Recommendation:

Kinase inhibitors targeting each oncogene are weakly recommended for squamous cell lung cancer patients who have driver oncogene.

Recommendation: 2

Evidence level: D

Agreement rate 85%

Comments:

Among *EGFR-mutated*, squamous cell lung cancer patients, no comparative study has been conducted using EGFR-TKI. However, subgroup data from prospective trials and their pooled analysis were reported. In a prospective trial, 11 patients for whom data were available demonstrated ORR of 9.1%. Although this suggests limited efficacy of EGFR-TKI in this population, caution is advised, because at least three patients had *uncommon EGFR mutation* in this report [69–71]. Several retrospective analyses with small sample size showed ORR of 25–32% and mPFS of 1.4–3.9 months [69, 72, 73]. No prospective trial has been conducted in patients with *uncommon EGFR mutation, ALK rearrangement, ROS1 rearrangement*, or *BRAF mutation*.

Based on this evidence, efficacy of kinase inhibitors in squamous cell lung cancer patients who have driver oncogene is limited, but some responders existed. The guideline committee weakly recommends for kinase inhibitors in squamous cell lung cancer patients who have driver oncogene. We judged quality of evidence as D and strength of recommendation as 2. The results of voting by committee members to determine this recommendation are described below.

Involved members regarding this voting: sub-committee on chemotherapy (medical doctors, pharmacists, nurses, statisticians, and patients)

**Table:** 

Benefit with strong recommendation	Benefit with weak recommendation	Unable to determine recommendation	No benefit or risk with weak recommendation	No benefit or risk with strong recommendation
15% (4/26)	85% (22/26)	0	0	0

#### PD-L1 ≥ 50% (Fig. 10)

**Fig. 10:**
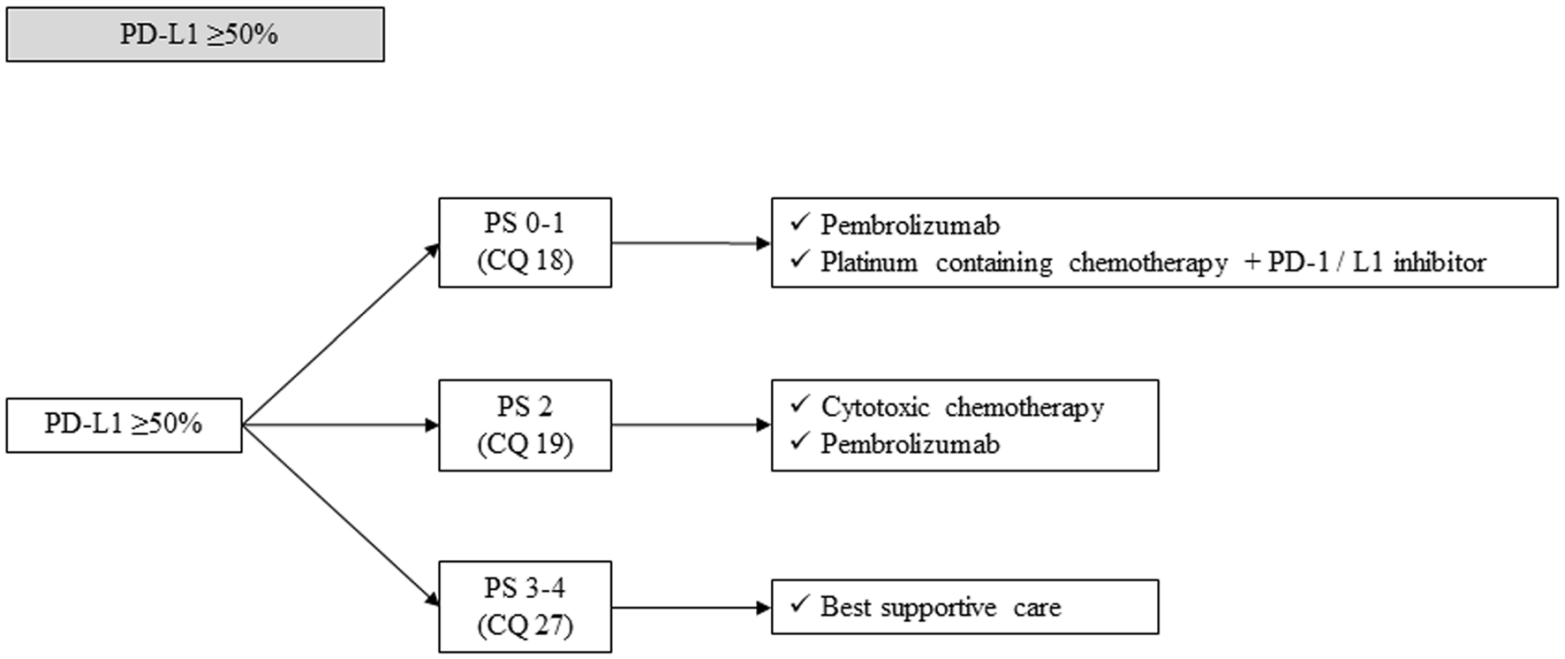
Treatment strategy in PD-L1 ≥ 50% NSCLC, stage IV. *PD-1* programed cell death-1, *PD-L1* programed death-ligand 1, *PS* performance status

##### CQ 18

What is the recommended first-line treatment in patients with PS 0–1 and whose tumor is positive for PD-L1 ≥ 50%?

Recommendation:

- Pembrolizumab monotherapy is strongly recommended.

Recommendation: 1

Evidence level: B

Agreement rate 96%

(b) Platinum-based chemotherapy plus PD-1/PD-L1 inhibitor is strongly recommended.

Recommendation: 1

Evidence level: B

Agreement rate 69%

Comments:

- A phase III trial comparing pembrolizumab with platinum-doublet chemotherapy was conducted in stage IV NSCLC patients with PS 0–1, whose tumor was positive for PD-L1 ≥ 50%, and who did not have *EGFR mutation* or *ALK rearrangement* (KEYNOTE-024 study) [74]. In this trial, 305 patients were randomized and 66 (43.7%) were treated with pembrolizumab after progression of platinum-doublet chemotherapy as a crossover treatment. In an interim analysis, PFS (primary endpoint) was significantly prolonged in pembrolizumab arm [10.3 months versus 6.0 months, HR 0.50 (95%CI: 0.37–0.68), *p* < 0.001]. OS (secondary endpoint) was also significantly prolonged [median not reached in either arm, HR 0.60 (95%CI: 0.41–0.89), *p* = 0.005]. In addition, ORR was significantly improved (44.8% versus 27.8%). Main adverse events were diarrhea, fatigue, and pyrexia in the pembrolizumab arm, while anemia, nausea, and fatigue were common in the platinum-doublet chemotherapy arm. There were fewer adverse events ≥ grade 3 in pembrolizumab arm (26.6% versus 53.3%). On the other hand, immune-related adverse events such as thyroid dysfunction, pneumonitis, skin rash, and colitis were reported (9.7% were ≥ grade 3). Careful management should be applied.
  Regarding the elderly patients ≥ 75 years old, no subgroup analysis from this trial or any prospective data has been reported.
  Based on this evidence, pembrolizumab monotherapy is strongly recommended in patients whose tumor is positive for PD-L1 ≥ 50% and does not have *EGFR mutation* or *ALK rearrangement*. We judged quality of evidence as B and strength of recommendation as 1. The results of voting by committee members to determine this recommendation are described below.

Involved members regarding this voting: sub-committee on chemotherapy (medical doctors, pharmacists, nurses, statisticians, and patients)

**Table:** 

Benefit with strong recommendation	Benefit with weak recommendation	Unable to determine recommendation	No benefit or risk with weak recommendation	No benefit or risk with strong recommendation
96% (26/27)	4% (1/27)	0	0	0

1. Non-squamous cell lung cancer.

A phase III trial comparing platinum-doublet chemotherapy plus pembrolizumab with platinum-doublet chemotherapy was conducted in stage IV, non-squamous NSCLC patients with PS 0–1 and who did not have *EGFR mutation* or *ALK rearrangement* (KEYNOTE-189 study) [20]. In this trial, 616 patients were randomized in 2:1 ratio and 67 (32.5%) were treated with pembrolizumab monotherapy after progression of platinum-doublet chemotherapy as a crossover treatment. In an interim analysis, PFS (primary endpoint) was significantly prolonged in chemotherapy plus pembrolizumab arm [8.8 months versus 4.9 months, HR 0.52 (95%CI: 0.43–0.64), *p* < 0.0001]. OS (secondary endpoint) was also significantly prolonged [median not reached versus 11.3 months, HR 0.49 (95%CI: 0.38–0.64), *p* < 0.0001]. In a subset analysis of PD-L1 ≥ 50%, both PFS and OS were prolonged [PFS: 9.4 months versus 4.7 months, HR 0.36 (95%CI: 0.25–0.52), *p* < 0.0001; and OS: median not reached versus 10.0 months, HR 0.42 (95%CI: 0.26–0.68), *p* = 0.0001]. Main adverse events were nausea, anemia, fatigue, and constipation in chemotherapy plus pembrolizumab arm, and adverse events ≥ grade 3 were similar (67.2% versus 65.8%). On the other hand, in chemotherapy plus pembrolizumab arm, acute kidney injury and immune-related adverse events ≥ grade 3 were observed in 5.2% and 8.9% of the patients, respectively. In addition, three treatment-related deaths due to interstitial lung disease were reported. Careful management should be applied.

Another phase III trial, comparing platinum-based chemotherapy plus atezolizumab with platinum-based chemotherapy, was conducted in stage IV, non-squamous NSCLC patients with PS 0–1 (IMPOWER150 study). Results of CBDCA + PTX + bevacizumab plus atezolizumab (arm B) and CBDCA + PTX + bvacizumab (arm C) were reported [21]. PFS (one of the co-primary endpoints) was significantly prolonged in chemotherapy plus atezolizumab arm [8.3 months versus 6.8 months, HR 0.62 (95%CI: 0.52–0.74), *p* < 0.001]. OS (another co-primary endpoint) was also significantly prolonged [19.2 months versus 14.7 months, HR 0.78 (95%CI: 0.64-96), *p* = 0.02]. In a subset analysis of PD-L1 with TC3 or IC3, both PFS and OS were prolonged [PFS: 12.6 months versus 6.8 months, HR 0.39 (95%CI: 0.25–0.60), *p* < 0.0001; and OS: 25.2 months versus 15.0 months, HR 0.70 (95%CI: 0.43–1.13)]. Main adverse events were appetite loss, peripheral neuropathy, nausea, and fatigue in chemotherapy plus atezolizumab arm, and adverse events ≥ grade 3 were slightly higher (58.5% versus 50.0%). In chemotherapy plus atezolizumab arm, immune-related adverse events such as skin rash, liver dysfunction, thyroid dysfunction, pneumonitis, and colitis were reported. Careful management should be applied.

(b-2) Squamous cell lung cancer.

A phase III trial comparing platinum-doublet chemotherapy plus pembrolizumab with platinum-doublet chemotherapy was conducted in stage IV, squamous cell lung cancer patients with PS 0–1 (KEYNOTE-407 study) [22]. In this trial, 559 patients were randomized. In an interim analysis, PFS (one of the co-primary endpoints) was significantly prolonged in chemotherapy plus pembrolizumab arm [6.4 months versus 4.8 months, HR 0.56 (95%CI: 0.45–0.70), *p* < 0.0001]. OS (another primary endpoint) was also significantly prolonged [15.9 months versus 11.3 months, HR 0.64 (95%CI: 0.49–0.85), *p* = 0.0008]. In a subset analysis of PD-L1 ≥ 50%, both PFS and OS tended to be prolonged [PFS: 8.0 months versus 4.2 months, HR 0.37 (95%CI: 0.24–0.58); and OS: median not reached in either arm, HR 0.64 (95%CI: 0.37–1.10)]. Main adverse events were anemia, appetite loss, neutropenia, and nausea in chemotherapy plus pembrolizumab arm, and adverse events ≥ grade 3 were similar (69.8% versus 68.2%). On the other hand, treatment-related deaths were higher in chemotherapy plus pembrolizumab arm (3.6% versus 2.1%).

Another phase III trial, comparing platinum-doublet chemotherapy plus atezolizumab with platinum-doublet chemotherapy, was conducted in stage IV, squamous cell lung cancer patients with PS 0–1 (IMPOWER131 study). Results of CBDCA + nab-PTX plus atezolizumab (arm B) and CBDCA + nab-PTX (arm C) were reported [23]. PFS (one of the co-primary endpoints) was significantly prolonged in chemotherapy plus atezolizumab arm [6.3 months versus 5.6 months, HR 0.71 (95%CI: 0.60–0.85), *p* = 0.001]. However, improvement of OS (another co-primary endpoint) was not shown at the time of this interim analysis [14.0 months versus 13.9 months, HR 0.96 (95%CI: 0.78–1.18), *p* = 0.6931]. In a subset analysis of PD-L1 with TC3 or IC3, both PFS and OS were prolonged [PFS: 10.1 months versus 5.5 months, HR 0.44 (95%CI: 0.27–0.71); and OS: 23.6 months versus 14.1 months, HR 0.56 (95%CI: 0.32–0.99)]. Adverse events ≥ grade 3 were higher (69% versus 58%) in the chemotherapy plus atezolizumab arm.

Regarding the elderly patients ≥ 75 years old, these four trials [20–23] allowed registration of this population. In two trials using atezolizumab, subgroup analyses were reported. Both trials showed that PFS was better in the chemotherapy plus atezolizumab arm, even in the elderly [9.7 months versus 6.8 months (HR 0.78) in the IMpower150 study and 7.0 months versus 5.6 months (HR 0.78) in the IMpower131 study] [21, 23]. However, safety data were not reported in this population. Careful management should be applied.

Based on this evidence, platinum-based chemotherapy plus PD-1/PD-L1 inhibitor is strongly recommended in patients with PS 0–1, whose tumor is positive for PD-L1 ≥ 50%, and who do not have *EGFR mutation* or *ALK rearrangement*. On the other hand, as there has been no direct comparison between chemotherapy plus PD-1/PD-L1 inhibitor and pembrolizumab alone, the superiority has not been clarified. We judged quality of evidence as B and strength of recommendation as 1. The results of voting by committee members to determine this recommendation are described below.

Involved members regarding this voting: sub-committee on chemotherapy (medical doctors, pharmacists, nurses, statisticians, and patients)

**Table:** 

Benefit with strong recommendation	Benefit with weak recommendation	Unable to determine recommendation	No benefit or risk with weak recommendation	No benefit or risk with strong recommendation
69% (18/26)	31% (8/26)	0	0	0

##### CQ 19

What is the recommended first-line treatment in patients with PS 2 and whose tumor is positive for PD-L1 ≥ 50%?

Recommendation:

- Cytotoxic chemotherapy is strongly recommended.

Monotherapy;

Recommendation: 1

Evidence level: A

Agreement rate xx%

CBDCA—doublet therapy;

Recommendation: 2

Evidence level: B

Agreement rate xx%

(b) Pembrolizumab monotherapy is weakly recommended.

Recommendation: 2

Evidence level: D

Agreement rate 85%

(c) There is no clear evidence to recommend the combination of platinum-based chemotherapy plus PD-1/PD-L1 inhibitor.

Unable to determine recommendation.

Comments:

- Refer to CQ 22.
- As the KEYNOTE-024 study allowed enrollment of patients with PS 0–1 [74], there have been no efficacy or safety data of pembrolizumab monotherapy in stage IV NSCLC patients with PS 2. On the other hand, there is limited evidence regarding cytotoxic chemotherapy in this area. The efficacy was modest, while these patients often suffered from adverse events. Regarding the lesser toxicity of pembrolizumab, many guideline committee members supported pembrolizumab as an option for this population.
  Based on this evidence, after careful consideration, the guideline committee weakly recommends the administration of pembrolizumab monotherapy in patients with PS 2 and whose tumor is positive for PD-L1 ≥ 50% as an expert opinion. We judged quality of evidence as D and strength of recommendation as 2. The results of voting by committee members to determine this recommendation are described below.

Involved members regarding this voting: sub-committee on chemotherapy (medical doctors, pharmacists, nurses, statisticians, and patients)

**Table:** 

Benefit with strong recommendation	Benefit with weak recommendation	Unable to determine recommendation	No benefit or risk with weak recommendation	No benefit or risk with strong recommendation
0	85% (22/26)	15% (4/26)	0	0

(c) As four phase III trials comparing platinum-based chemotherapy plus PD-1/PD-L1 inhibitor with platinum-based chemotherapy allowed enrollment of patients with PS 0–1, there are no efficacy or safety data of platinum-based chemotherapy plus PD-1/PD-L1 inhibitor in stage IV NSCLC patients with PS 2. Basically, there are some safety concerns regarding cytotoxic chemotherapy in this population. Thus, adding PD-1/PD-L1 inhibitor to platinum-based chemotherapy may not be tolerable.
  Based on this, there is no clear evidence to recommend the combination of platinum-based chemotherapy plus PD-1/PD-L1 inhibitor in patients with PS 2 and whose tumor is positive for PD-L1 ≥ 50%. The guideline committee finally determined that there is insufficient evidence to draw a firm conclusion on this CQ. The results of voting by committee members to determine this recommendation are described below.

Involved members regarding this voting: sub-committee on chemotherapy (medical doctors, pharmacists, nurses, statisticians, and patients)

**Table:** 

Benefit with strong recommendation	Benefit with weak recommendation	Unable to determine recommendation	No benefit or risk with weak recommendation	No benefit or risk with strong recommendation
0	0	69% (18/26)	27% (7/26)	4% (1/26)

### Driver oncogene-negative and PD-L1 < 50%, or unknown

#### First-line treatment in patients who are driver oncogene-negative and PD-L1 < 50%, or unknown is shown in Fig. 11

**Fig. 11:**
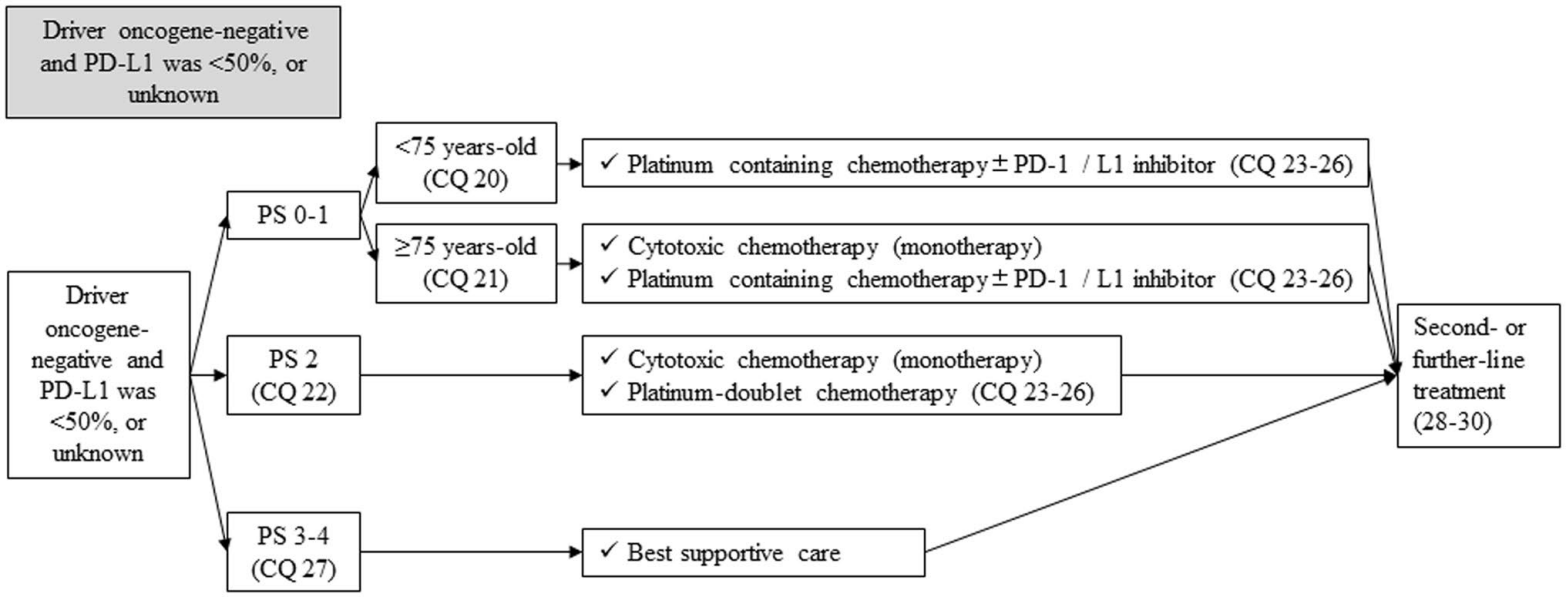
First-line treatment in patients with stage IV NSCLC, who were driver oncogene-negative and PD-L1 was < 50%, or unknown. *PD-1* programed cell death-1, *PD-L1* programed death-ligand 1, *PS* performance status

##### CQ 20

Is cytotoxic chemotherapy recommended as a first-line treatment in patients with PS 0–1 and younger than 75 years old, when their tumor is driver oncogene-negative and PD-L1 is < 50%, or unknown?

Recommendation:

Combination of platinum agent and third- or later-generation drug is strongly recommended.

Recommendation: 1

Evidence level: A

Agreement rate 93%

Comments:

A meta-analysis demonstrated that platinum-based chemotherapy significantly prolonged OS compared with BSC [1]. In another meta-analysis, comparing second-generation agent with third-generation agent as a part of a platinum-based regimen, the latter was found to be superior, with ORR of 12% and 1-year survival rate of 6% [2]. In a Japanese phase III trial (FACS study), four regimens of platinum agents plus third-generation agents were compared, and their efficacies were similar [75].

Newer agents demonstrated the efficacy in several phase III studies, but some showed their efficacy on specific histology types. PEM is one typical example and is approved for non-squamous cell lung cancer. In study JMDB, comparing cisplatin (CDDP) + PEM with CDDP + gemcitabine (GEM), efficacy results were similar in overall population. However, CDDP + PEM showed better OS in non-squamous cell lung cancer [11.8 versus 10.4 months, HR 0.81 (95%CI: 0.70–0.94), *p* = 0.005], while inferior OS was shown in squamous cell lung cancer [9.4 months versus 10.8 months, HR 1.23 (95%CI: 1.00–1.51), *p* = 0.05] [76]. Even though this was a subset analysis, CDDP + PEM are considered one of the preferred regimens for non-squamous cell lung cancer in terms of efficacy and safety. Regarding CBDCA + PEM, although there has been no phase III trial to investigate its OS benefit, it is commonly used due to its milder non-hematologic toxicity than CDDP. In randomized studies comparing CBDCA + PEM with CBDCA + GEM, CBDCA + DTX, or CBDCA + PTX + bevacizumab, superiority in OS or safety was not demonstrated [77–79]. On the other hand, Kaplan–Meier curves of OS seemed to be similar between CBDCA + PEM and CBDCA + PTX + bevacizumab [79], and PFS of CBDCA + PEM + bevacizumab tended to be better than CBDCA + PTX + bevacizumab [80]. Based on these results, CBDCA + PEM can be considered as a first-line treatment regimen.

For squamous cell lung cancer, a Japanese phase III trial comparing CDGP + DTX with CDDP + DTX was conducted [81]. OS was significantly prolonged in CDGP arm [13.6 months versus 11.4 months, HR 0.81 (95%CI: 0.65–1.02), *p* = 0.037]. Regarding toxicity, leukopenia, neutropenia, and platelet count decrease were common in CDGP arm, while nausea, fatigue, hyponatremia, and hypokalemia were common in CDDP arm. This was the only regimen to show OS superiority in Japan.

In other phase III trials of S-1, non-superiority was demonstrated comparing CBDCA + S-1 with CBDCA + PTX against CDDP + S-1 with CDDP + DTX [82, 83]. In a phase III trial comparing CBDCA + nab-PTX with CBDCA + PTX, significant ORR improvement was demonstrated [33.0% versus 25.0 months, response rate ratio 1.31 (95%CI: 1.08–1.59), *p* = 0.005] [84]. These regimens can be used regardless of histology.

Based on this evidence, combination of platinum agent and third- or later-generation drug is strongly recommended in patients with PS 0–1 and younger than 75 years old. We judged quality of evidence as A and strength of recommendation as 1. The results of voting by committee members to determine this recommendation are described below.

Involved members regarding this voting: sub-committee on chemotherapy (medical doctors, pharmacists, nurses, statisticians, and patients)

**Table:** 

Benefit with strong recommendation	Benefit with weak recommendation	Unable to determine recommendation	No benefit or risk with weak recommendation	No benefit or risk with strong recommendation
93% (25/27)	7% (2/27)	0	0	0

##### CQ 21

Is cytotoxic chemotherapy recommended as a first-line treatment in patients with PS 0–1 and older than 75 years old, when their tumor is driver oncogene-negative and PD-L1 is < 50%, or unknown?

Recommendation:

- Monotherapy using third-generation drug is strongly recommended.

Recommendation: 1

Evidence level: A

Agreement rate 100%

(b) CBDCA doublet chemotherapy is weakly recommended.

Recommendation: 2

Evidence level: B

Agreement rate 74%

Comments:

In a post hoc analysis of a phase III trial of first-line treatment and adjuvant chemotherapy, efficacy was similar between those who were younger than 65 years and who were 65 years of age or older [85]. They also reported that instrumental ADL, not chronological age, was associated with better prognosis. Another report mentioned that octogenarian patients did not show worse OS than younger patients (7 months versus 11 months, *p* = 0.20) and they also had similar safety result, even when they had PS of 0–1 [86]. These reports suggest that elderly patients should not be excluded from anti-cancer treatment due to their chronological age.

- Phase III trials demonstrated that vinorelbine (VNR) prolonged OS compared with BSC, and also showed that GEM had similar efficacy to VNR [87, 88]. In a Japanese phase III trial (WJTOG9904 study) comparing DTX with VNR, PFS was significantly prolonged in DTX arm [5.5 months versus 3.1 months, HR 0.61 (95%CI: 0.45–0.82), *p* < 0.001]. OS was favorable in DTX arm, but without statistical significance [14.3 months versus 9.9 months, HR 0.78 (95%CI: 0.56–1.09), *p* = 0.138] [89].

Based on this evidence, monotherapy using a third-generation drug is strongly recommended in patients with PS 0–1 and older than 75 years old. We judged quality of evidence as A and strength of recommendation as 1. The results of voting by committee members to determine this recommendation are described below.

Involved members regarding this voting: sub-committee on chemotherapy (medical doctors, pharmacists, nurses, statisticians, and patients)

**Table:** 

Benefit with strong recommendation	Benefit with weak recommendation	Unable to determine recommendation	No benefit or risk with weak recommendation	No benefit or risk with strong recommendation
100% (27/27)	0	0	0	0

(b) For elderly NSCLC patients, two phase III trials comparing platinum-doublet chemotherapy with third-generation drug monotherapy were reported, and most of the participants were ≥ 75 years old. Of those, the Japanese study (JCOG-0803/WJOG-4307L) compared weekly CDDP + DTX with DTX [90]. Interim analysis showed that combination treatment was not superior to monotherapy in OS [13.3 months versus 14.8 months, HR 1.18 (95% CI: 0.83–1.69)], which resulted in early termination. Study IFCT0501 compared CBDCA + weekly PTX with GEM or VNR [91]. PFS and OS were significantly prolonged in combination arm [PFS: 6.0 months versus 2.8 months, HR 0.51 (95% CI: 0.42–0.62), *p* < 0.001; and OS: 10.3 months versus 6.2 months, HR 0.64 (95%CI: 0.52–0.78), *p* < 0.0001]. However, these results were not more satisfactory than DTX monotherapy in a Japanese trial, and treatment-related deaths were relatively high in combination arm (4.4%). In addition, dosing of combination arm was unfamiliar in Japanese clinical practice. These results should, therefore, be interpreted with caution.

Based on this evidence, CBDCA doublet chemotherapy is weakly recommended in patients with PS 0–1 and older than 75 years old. We judged quality of evidence as A and strength of recommendation as 1. The results of voting by committee members to determine this recommendation are described below.

Involved members regarding this voting: sub-committee on chemotherapy (medical doctors, pharmacists, nurses, statisticians, and patients)

**Table:** 

Benefit with strong recommendation	Benefit with weak recommendation	Unable to determine recommendation	No benefit or risk with weak recommendation	No benefit or risk with strong recommendation
26% (7/27)	74% (20/27)	0	0	0

##### CQ 22

Is cytotoxic chemotherapy recommended as a first-line treatment in patients with PS 2, when their tumor is driver oncogene-negative and PD-L1 is < 50%, or unknown?

Recommendation:

- Monotherapy using third-generation drug is strongly recommended.

Recommendation: 1

Evidence level: A

Agreement rate 100%

(b) Platinum-doublet chemotherapy is weakly recommended.

Recommendation: 2

Evidence level: B

Agreement rate 100%

Comments:

Because PS 2 comprises heterogeneous patients, standard treatment has not been established. In a subset of a meta-analysis comparing chemotherapy with BSC, OS was prolonged by chemotherapy regardless of PS [6% improvement in 1-year OS rate in patients with PS ≥ 2 (14% versus 8%)] [1].

- A meta-analysis comparing the third-generation drugs (DTX, PTX, VNR, or GEM) with BSC demonstrated 7% of improvement in 1-year OS rate [2]. In this study, about 30% of patients had PS 2. From the three trials that were included in this meta-analysis, subset analyses of patients with PS 2 were reported, and each of the OS results tended to be prolonged [92].
  Based on this evidence, monotherapy using a third-generation drug is strongly recommended in patients with PS 2. We judged quality of evidence as A and strength of recommendation as 2. The results of voting by committee members to determine this recommendation are described below.

Involved members regarding this voting: sub-committee on chemotherapy (medical doctors, pharmacists, nurses, statisticians, and patients)

**Table:** 

Benefit with strong recommendation	Benefit with weak recommendation	Unable to determine recommendation	No benefit or risk with weak recommendation	No benefit or risk with strong recommendation
100% (27/27)	0	0	0	0

(b) A phase III trial comparing CBDCA + PTX with PTX (CALGB9730 study), subset analysis of PS 2 showed that 1-year survival rate was improved in the combination arm [18% versus 10%, HR 0.60 (95%CI: 0.40–0.91), *p* = 0.016] [93]. In ECOG1599 study comparing CBDCA + PTX with CDDP + GEM in patients with PS 2, OS was 6.2 months and 6.9 months, respectively, and these regimens were considered as feasible [94]. In a randomized phase II trial comparing CBDCA + GEM with GEM, OS and PFS tended to be longer [OS: 6.7 months versus 4.8 months (*p* = 0.49), PFS: 4.1 months versus 3.0 months (*p* = 0.36)] [95]. Recently, a phase III trial comparing CBDCA + PEM with PEM was conducted [96]. This study had some limitations: only 205 patients were enrolled, which was relatively small as a phase III trial, and the inclusion of squamous cell lung cancer was allowed. However, OS was significantly improved in the combination arm [9.3 months versus 5.3 months, HR 0.62 (95%CI: 0.46–0.83), *p* = 0.001]. PFS was also improved [5.8 months versus 2.8 months, HR 0.46 (95%CI: 0.35–0.63), *p* < 0.001]. Regarding toxicity, anemia and neutropenia were common in the combination arm and treatment-related death was observed in 3.9% of the patients.
  Based on this evidence, platinum-doublet chemotherapy is weakly recommended in patients with PS 2. We judged quality of evidence as B and strength of recommendation as 2. The guideline committee calls attention to the clinicians that this evidence is limited, and most regimens in these trials were CBDCA-based or reduced-dosed. The results of voting by committee members to determine this recommendation are described below.

Involved members regarding this voting: sub-committee on chemotherapy (medical doctors, pharmacists, nurses, statisticians, and patients)

**Table:** 

Benefit with strong recommendation	Benefit with weak recommendation	Unable to determine recommendation	No benefit or risk with weak recommendation	No benefit or risk with strong recommendation
0	100% (27/27)	0	0	0

##### CQ 23

Is addition of PD-1/PD-L1 inhibitor to platinum-based chemotherapy recommended in patients with PS 0–2, when their tumor is driver oncogene-negative and PD-L1 is < 50%, or unknown?

Recommendation:

- Addition of PD-1/PD-L1 inhibitor to platinum-based chemotherapy is strongly recommended in patients with PS 0–1.

Recommendation: 1

Evidence level: B

Agreement rate 78%

(b) There is no clear evidence to recommend the addition of PD-1/PD-L1 inhibitor to platinum- containing chemotherapy in patients with PS 2.

Unable to determine recommendation.

Comments:

1. Non-squamous cell lung cancer.

A phase III trial comparing platinum-doublet chemotherapy plus pembrolizumab with platinum-doublet chemotherapy was conducted in stage IV, non-squamous NSCLC patients with PS 0–1 and who did not have *EGFR mutation* or *ALK rearrangement* (KEYNOTE-189 study) [20]. 616 patients were randomized in 2:1 ratio and 67 (32.5%) were treated with pembrolizumab monotherapy after progression of platinum-doublet chemotherapy as a crossover treatment. In an interim analysis, PFS (primary endpoint) was significantly prolonged in chemotherapy plus pembrolizumab arm [8.8 months versus 4.9 months, HR 0.52 (95%CI: 0.43–0.64), *p* < 0.0001]. OS (secondary endpoint) was also significantly prolonged [median not reached versus 11.3 months, HR 0.49 (95%CI: 0.38–0.64), *p* < 0.0001]. In a subset analysis of PD-L1 1–49%, both PFS and OS were prolonged [PFS: 9.0 months versus 4.9 months, HR 0.55 (95%CI: 0.37–0.81); and OS: median not reached versus 12.9 months, HR 0.55 (95%CI: 0.34–0.90)]. In patients with PD-L1 < 1%, both PFS and OS were prolonged [PFS: 6.1 months versus 5.1 months, HR 0.75 (95%CI: 0.53–1.05); and OS: 15.2 months versus 12.0 months, HR 0.59 (95%CI: 0.38–0.92)]. Main adverse events were nausea, anemia, fatigue, and constipation in chemotherapy plus pembrolizumab arm, and adverse events ≥ grade 3 were similar (67.2% versus 65.8%). On the other hand, in chemotherapy plus pembrolizumab arm, acute kidney injury and immune-related adverse events ≥ grade 3 were observed in 5.2% and 8.9% of the patients, respectively. In addition, three treatment-related deaths due to interstitial lung disease were reported. Careful management should be applied.

Another phase III trial comparing platinum-based chemotherapy plus atezolizumab with platinum-based chemotherapy was conducted in stage IV, non-squamous NSCLC patients with PS 0–1 (IMPOWER150 study). Results of CBDCA + PTX + bevacizumab plus atezolizumab (arm B) and CBDCA + PTX + bevacizumab (arm C) were reported [21] PFS (one of co-primary endpoints) was significantly prolonged in chemotherapy plus atezolizumab arm [8.3 months versus 6.8 months, HR 0.62 (95%CI: 0.52–0.74), *p* < 0.001]. OS (another co-primary endpoint) was also significantly prolonged [19.2 months versus 14.7 months, HR 0.78 (95%CI: 0.64-96), *p* = 0.02]. In a subset analysis of PD-L1 with TC1/2 or IC1/2, both PFS and OS were prolonged [PFS: 8.3 months versus 6.6 months, HR 0.56 (95%CI: 0.41–0.77); and OS: 20.3 months versus 16.4 months, HR 0.80 (95%CI: 0.55–1.15)]. In patients with PD-L1 with TC0 and IC0, both PFS and OS were prolonged [PFS: 7.1 months versus 6.9 months, HR 0.77 (95%CI: 0.61–0.99); and OS: 17.1 months versus 14.1 months, HR 0.82 (95%CI: 0.62–1.08)]. Main adverse events were appetite loss, peripheral neuropathy, nausea, and fatigue in chemotherapy plus atezolizumab arm, and adverse events ≥ grade 3 were slightly higher (58.5% versus 50.0%). In chemotherapy plus atezolizumab arm, immune-related adverse events such as skin rash, liver dysfunction, thyroid dysfunction, pneumonitis, and colitis were reported. Careful management should be applied.

(a-2) Squamous cell lung cancer.

A phase III trial comparing platinum-doublet chemotherapy plus pembrolizumab with platinum-doublet chemotherapy was conducted in stage IV squamous cell lung cancer patients with PS 0–1 (KEYNOTE-407 study) [22]. In this trial, 559 patients were randomized. In an interim analysis, PFS (one of the co-primary endpoints) was significantly prolonged in chemotherapy plus pembrolizumab arm [6.4 months versus 4.8 months, HR 0.56 (95%CI: 0.45–0.70), *p* < 0.0001]. OS (another primary endpoint) was also significantly prolonged [15.9 months versus 11.3 months, HR 0.64 (95%CI: 0.49–0.85), *p* = 0.0008]. In a subset analysis of PD-L1 1–49%, both PFS and OS were prolonged [PFS: 7.2 months versus 5.2 months, HR 0.56 (95%CI: 0.39–0.80); and OS: 14.0 months versus 11.6 months, HR 0.57 (95%CI: 0.57–0.90)]. In patients with PD-L1 < 1%, both PFS and OS were prolonged [PFS: 6.3 months versus 5.3 months, HR 0.68 (95%CI: 0.47–0.98); and OS: 15.9 months versus 10.2 months, HR 0.61 (95%CI: 0.38–0.98)]. Main adverse events were anemia, appetite loss, neutropenia, and nausea in chemotherapy plus pembrolizumab arm, and adverse events ≥ grade 3 were similar (69.8% versus 68.2%). On the other hand, treatment-related deaths were higher in chemotherapy plus pembrolizumab arm (3.6% versus 2.1%).

Another phase III trial, comparing platinum-doublet chemotherapy plus atezolizumab with platinum-doublet chemotherapy, was conducted in stage IV, squamous cell lung cancer patients with PS 0–1 (IMPOWER131 study). Results of CBDCA + nab-PTX plus atezolizumab (arm B) and CBDCA + nab-PTX (arm C) were reported [23]. PFS (one of the co-primary endpoints) was significantly prolonged in chemotherapy plus atezolizumab arm [6.3 months versus 5.6 months, HR 0.71 (95%CI: 0.60–0.85), *p* = 0.001]. However, improvement of OS (another co-primary endpoint) was not shown at the time of this interim analysis [14.0 months versus 13.9 months, HR 0.96 (95%CI: 0.78–1.18), *p* = 0.6931]. In a subset analysis of PD-L1 with TC1/2 or IC1/2, PFS tended to be longer, but OS was not prolonged [PFS: 6.0 months versus 5.6 months, HR 0.70(95%CI: 0.53–0.92); and OS: 12.4 months versus 16.6 months, HR 1.34 (95%CI: 0.95–1.90)]. In patients with PD-L1 with TC0 and IC0, results were similar in PFS and OS [PFS: 5.7 months versus 5.6 months, HR 0.81 (95%CI: 0.64–1.03); and OS: 13.8 months versus 12.5 months, HR 0.86 (95%CI: 0.65–1.15)]. Adverse events ≥ grade 3 were higher (69% versus 58%) in the chemotherapy plus atezolizumab arm.

Regarding elderly patients ≥ 75 years old, these four trials [2, 75–77] allowed registration of this population. In two trials using atezolizumab, subgroup analyses were reported. Both trials showed that PFS was better in the chemotherapy plus atezolizumab arm, even in the elderly [9.7 months versus 6.8 months (HR 0.78) in the IMpower150 study; and 7.0 months versus 5.6 months (HR 0.78) in the IMpower131 study] [75, 77]. However, safety data were not reported in this population. Careful management should be applied.

Based on this evidence, addition of PD-1/PD-L1 inhibitor to platinum- containing chemotherapy is strongly recommended in patients with PS 0–1 when their tumor is driver oncogene-negative and PD-L1 is < 50%, or unknown. We judged quality of evidence as B and strength of recommendation as 1.

Involved members regarding this voting: sub-committee on chemotherapy (medical doctors, pharmacists, nurses, statisticians, and patients)

**Table:** 

Benefit with strong recommendation	Benefit with weak recommendation	Unable to determine recommendation	No benefit or risk with weak recommendation	No benefit or risk with strong recommendation
78% (21/27)	22% (6/27)	0	0	0

(b) As four phase III trials comparing platinum-based chemotherapy plus PD-1/PD-L1 inhibitor with platinum-based chemotherapy allowed enrollment of patients with PS 0–1 [20–23], there has been no efficacy or safety data of platinum-based chemotherapy plus PD-1/PD-L1 inhibitor in stage IV NSCLC patients with PS 2. Basically, there are some safety concerns regarding cytotoxic chemotherapy in this population. Thus, adding PD-1/PD-L1 inhibitor to platinum-based chemotherapy may not be tolerable.
  Based on this, there is no clear evidence to recommend the addition of PD-1/PD-L1 inhibitor to platinum-based chemotherapy in patients with PS 2, when their tumor is driver oncogene-negative and PD-L1 is < 50%, or unknown. The guideline committee finally determined that there is insufficient evidence to draw a firm conclusion on this CQ. The results of voting by committee members to determine this recommendation are described below.

Involved members regarding this voting: sub-committee on chemotherapy (medical doctors, pharmacists, nurses, statisticians, and patients)

**Table:** 

Benefit with strong recommendation	Benefit with weak recommendation	Unable to determine recommendation	No benefit or risk with weak recommendation	No benefit or risk with strong recommendation
0	0	74% (20/27)	22% (6/27)	4% (1/27)

##### CQ 24

What is the recommended number of courses of platinum-based chemotherapy?

Recommendation:

Six or fewer courses of platinum-based chemotherapy are strongly recommended.

Recommendation: 1

Evidence level: C

Agreement rate 100%

Comments:

Two phase III trials comparing three or four courses of combination chemotherapy consisting of platinum and third-generation agent with six courses of the same regimen both showed that 1-year survival and OS were similar, while toxicity was milder with the former [97, 98]. An individual-patient data meta-analysis that containing these two studies compared six courses with five or fewer courses demonstrated that PFS was significantly prolonged by six courses of treatment, but OS was similar [99].

Recent phase III trials mostly defined the maximum number of courses of platinum-based chemotherapy as four to six. In a phase III trial comparing CDDP + PEM with CDDP + GEM (JMDB study), the median number of courses of CDDP treatment was five in both arms [76].

Based on this evidence, six or fewer courses of platinum-based chemotherapy are strongly recommended. We judged quality of evidence as C and strength of recommendation as 1.

Involved members regarding this voting: sub-committee on chemotherapy (medical doctors, pharmacists, nurses, statisticians, and patients)

**Table:** 

Benefit with strong recommendation	Benefit with weak recommendation	Unable to determine recommendation	No benefit or risk with weak recommendation	No benefit or risk with strong recommendation
100% (26/26)	0	0	0	0

##### CQ 25

Is addition of bevacizumab to platinum-doublet chemotherapy recommended in patients with PS 0–2?

Recommendation:

- Addition of bevacizumab to platinum-doublet chemotherapy is weakly recommended in patients with PS 0–1 and < 75 years old.

Recommendation: 2

Evidence level: A

Agreement rate 73%

(b) Not to add bevacizumab to platinum-doublet chemotherapy is weakly recommended in patients ≥ 75 years old.

Recommendation: 2

Evidence level: C

Agreement rate 92%

(c) Not to add bevacizumab to platinum-doublet chemotherapy is weakly recommended in patients with PS 2.

Recommendation: 2

Evidence level: D

Agreement rate 96%

Comments:

- Meta-analyses demonstrated that addition of bevacizumab to platinum-doublet chemotherapy brought ORR improvement and prolongation of PFS. In addition, one of the analyses reported prolongation of OS [100, 101]. On the other hand, toxicity ≥ grade 3, such as proteinuria, hypertension, hemorrhagic events, neutropenia, febrile neutropenia, and treatment-related death, were significantly increased by adding bevacizumab [100–102].
  In a phase III trial comparing bevacizumab and CBDCA + PTX with CBDCA + PTX (ECOG4599 study), prolongation of OS and PFS was demonstrated [PFS: 6.2 months versus 4.5 months, HR 0.66 (95%CI: 0.57–0.77), *p* < 0.001; and OS: 12.3 months versus 10.3 months, HR 0.79 (95%CI: 0.67–0.92), *p* = 0.003] [103]. ORR was also improved. On the other hand, in another phase III trial comparing bevacizumab and CDDP + GEM with CDDP + GEM (AVAiL study), PFS was prolonged, but OS was similar [104]. In a Japanese phase II trial (JO19907 study) using the same regimen as in ECOG4599, ORR and PFS were better [ORR: 60.7% versus 31.0%, PFS: 6.9 months versus 5.9 months, HR 0.61 (95%CI 0.42–0.89), *p* = 0.009], but OS was not prolonged [22.8 months versus 23.4 months, HR 0.99 (0.65–1.50)] [105]. In China, a phase III trial (BEYOND study) also using the same regimen as in ECOG4599 was conducted [106]. That trial demonstrated prolongation of both PFS and OS [PFS: 9.2 months versus 6.5 months, HR 0.40 (95%CI: 0.29–0.54), *p* < 0.001; and OS: 24.3 months versus 17.7 months, HR 0.68 (95%CI: 0.50–0.93), *p* = 0.0154].
  Based on this evidence, addition of bevacizumab to platinum-doublet chemotherapy is weakly recommended in patients with PS 0–1 and < 75 years old. We judged quality of evidence as A and strength of recommendation as 2. The results of voting by committee members to determine this recommendation are described below.

Involved members regarding this voting: sub-committee on chemotherapy (medical doctors, pharmacists, nurses, statisticians, and patients)

**Table:** 

Benefit with strong recommendation	Benefit with weak recommendation	Unable to determine recommendation	No benefit or risk with weak recommendation	No benefit or risk with strong recommendation
27% (7/26)	73% (19/26)	0	0	0

(b) Elderly
  In a subset analysis of ECOG4599 study, the elderly population (> 70 years old) did not benefit from addition of bevacizumab, but showed increase of neutropenia, bleeding, and proteinuria ≥ grade 3 [107]. In another combined analysis, of ECOG4599 and the PointBreak study, benefits of OS and PFS were less, especially in patients who were > 75 years old [108]. In a retrospective study conducted in the US (ARIES study), efficacy was similar between patients age below 65, between 65 and 75, and those above 75 years, but arterial thrombosis ≥ grade 3 tended to be increased in the elderly subset (1.5% < 65 years, 2.9% ≥ 65 years, and 3.5% ≥ 75 years) [109]. In a cohort study conducted in Europe (SAiL study), efficacy was similar between patients aged below and above 70 years, but hemorrhagic events tended to be increased in the elderly subset (3.5% < 70 years, 5.3% ≥ 70 years). In Japan, there are no sufficient data of bevacizumab-containing chemotherapy in the elderly.
  Based on this evidence, not to add bevacizumab to platinum-doublet chemotherapy is weakly recommended in patients ≥ 75 years old. We judged quality of evidence as C and strength of recommendation as 2. The results of voting by committee members to determine this recommendation are described below.

Involved members regarding this voting: sub-committee on chemotherapy (medical doctors, pharmacists, nurses, statisticians, and patients)

**Table:** 

Benefit with strong recommendation	Benefit with weak recommendation	Unable to determine recommendation	No benefit or risk with weak recommendation	No benefit or risk with strong recommendation
0	0	4% (1/26)	96% (25/26)	0

(c) PS 2
  Regarding bevacizumab, as most of the clinical trials and observational studies consisted of patients with PS 0–1, there are few data regarding efficacy and toxicity in patients with PS 2 [109, 110]. Toxicity is significantly increased by adding bevacizumab, thus, not to add bevacizumab to platinum-doublet chemotherapy is weakly recommended in patients with PS 2. We judged quality of evidence as D and strength of recommendation as 2. The results of voting by committee members to determine this recommendation are described below.

Involved members regarding this voting: sub-committee on chemotherapy (medical doctors, pharmacists, nurses, statisticians, and patients)

**Table:** 

Benefit with strong recommendation	Benefit with weak recommendation	Unable to determine recommendation	No benefit or risk with weak recommendation	No benefit or risk with strong recommendation
0	0	4% (1/26)	92% (24/26)	4% (1/26)

##### CQ 26

Is maintenance therapy recommended in patients who received four courses of platinum-based chemotherapy without disease progression and with tolerable toxicity?

Recommendation:

Non-squamous cell lung cancer:

- In patients who received four courses of CDDP + PEM without disease progression and with tolerable toxicity, continuation maintenance with pemetrexed is strongly recommended.

Recommendation: 1

Evidence level: B

Agreement rate 100%

(b) In patients who received four courses of platinum-based chemotherapy without disease progression and with tolerable toxicity, switch maintenance with pemetrexed is weakly recommended.

Recommendation: 2

Evidence level: B

Agreement rate 88%

Squamous cell lung cancer:

(c) In patients who received four courses of platinum-based chemotherapy without disease progression and with tolerable toxicity, not to administer continuation or switch maintenance is strongly recommended.

Recommendation: 1

Evidence level: C

Agreement rate 100%

Note: maintenance therapy is a treatment strategy that is administered after multiple cycles of induction treatment with platinum-based regimen. Switch maintenance involves the introduction of a new agent. Continuation maintenance involves the continuation of an agent other than a platinum agent.

Comments:

- In a phase III trial of continuation maintenance with PEM after CDDP + PEM (PARAMOUNT study), PFS and OS were significantly prolonged [PFS: 4.1 months versus 2.8 months, HR 0.62 (95%CI: 0.50–0.73), *p* < 0.0001; and OS: 13.9 months versus 11.0 months, HR 0.78 (95%CI: 0.64–0.96), *p* = 0.0195] [112]. In the maintenance arm, QOL was not deteriorated. Toxicity was higher, but manageable. Another phase III trial compared maintenance PEM + bevacizumab with bevacizumab after CDDP + PEM + bevacizumab (AVAPERL study) [113]. In that trial, PFS was significantly prolonged [7.4 months versus 3.7 months, HR 0.48 (95%CI: 0.44–0.75), *p* < 0.0001], but OS was not significant.
  Based on this evidence, in patients who received four courses of CDDP + PEM without disease progression and with tolerable toxicity, continuation maintenance with pemetrexed is strongly recommended.
  We judged quality of evidence as B and strength of recommendation as 1. The results of voting by committee members to determine this recommendation are described below.

Involved members regarding this voting: sub-committee on chemotherapy (medical doctors, pharmacists, nurses, statisticians, and patients)

**Table:** 

Benefit with strong recommendation	Benefit with weak recommendation	Unable to determine recommendation	No benefit or risk with weak recommendation	No benefit or risk with strong recommendation
100% (26/26)	0	0	0	0

(b) In a phase III trial of switch maintenance with PEM after platinum-doublet chemotherapy, PFS and OS were significantly prolonged [PFS: 4.3 months versus 2.6 months, HR 0.50 (95%CI: 0.42–0.61), *p* < 0.0001; and OS: 13.4 months versus 10.6 months, HR 0.79 (95%CI: 0.65–0.95), *p* = 0.012] [114]. However, there was a limitation that crossover rate to PEM in the placebo arm was low (18%).
  Based on this evidence, in patients who received four courses of platinum-based chemotherapy without disease progression and with tolerable toxicity, switch maintenance with pemetrexed is weakly recommended. We judged quality of evidence as B and strength of recommendation as 2. The results of voting by committee members to determine this recommendation are described below.

Involved members regarding this voting: sub-committee on chemotherapy (medical doctors, pharmacists, nurses, statisticians, and patients)

**Table:** 

Benefit with strong recommendation	Benefit with weak recommendation	Unable to determine recommendation	No benefit or risk with weak recommendation	No benefit or risk with strong recommendation
12% (3/26)	88% (23/26)	0	0	0

(c) Phase III trials of switch maintenance with PEM or erlotinib demonstrated both PFS and OS prolongation, but OS prolongation was not shown in a squamous cell lung cancer subset [114, 115]. In another phase III trial, of switch maintenance with erlotinib (IUNO study), OS was not prolonged in the subset analysis of squamous cell lung cancer [9.7 months versus 9.5 months, HR 1.00 (95%CI: 0.74–1.35), *p* = 0.82] [116].
  Based on this evidence, in patients who received four courses of platinum-based chemotherapy without disease progression and with tolerable toxicity, not to administer continuation or switch maintenance is strongly recommended. We judged quality of evidence as C and strength of recommendation as 1. The results of voting by committee members to determine this recommendation are described below.

Involved members regarding this voting: sub-committee on chemotherapy (medical doctors, pharmacists, nurses, statisticians, and patients)

**Table:** 

Benefit with strong recommendation	Benefit with weak recommendation	Unable to determine recommendation	No benefit or risk with weak recommendation	No benefit or risk with strong recommendation
0	0	0	0	100% (25/25)

##### CQ 27

Is chemotherapy recommended in patients with PS 3–4, when their tumor is driver oncogene-negative or unknown?

Recommendation:

Not to administer any chemotherapy is strongly recommended.

Recommendation: 1

Evidence level: D

Agreement rate 100%

Comments:

Cytotoxic chemotherapy was usually not indicated in patients with PS 3–4. Regarding PD-1/PD-L1 inhibitor, clinical trials enrolled mainly patients with PS 0–1. Efficacy and safety data were unknown in patients with PS 3–4. In a phase III trial, erlotinib was compared with BSC in patients with poor PS or concomitant disease (TOPICAL study [117]). Median age of participants was 77, 30% had PS 3, and 52% were *EGFR wild-type*. OS (primary endpoint) was not prolonged [3.7 months versus 3.6 months, HR 0.94 (95%CI: 0.81–1.10), *p* = 0.46].

Based on this evidence, not to administer any chemotherapy is strongly recommended. We judged quality of evidence as D and strength of recommendation as 1.

Involved members regarding this voting: sub-committee on chemotherapy (medical doctors, pharmacists, nurses, statisticians, and patients)

**Table:** 

Benefit with strong recommendation	Benefit with weak recommendation	Unable to determine recommendation	No benefit or risk with weak recommendation	No benefit or risk with strong recommendation
0	0	0	0	100% (25/25)

#### Second- or further-line treatment in patients who are driver oncogene-negative and PD-L1 is < 50% or unknown (Fig. 12)

**Fig. 12:**
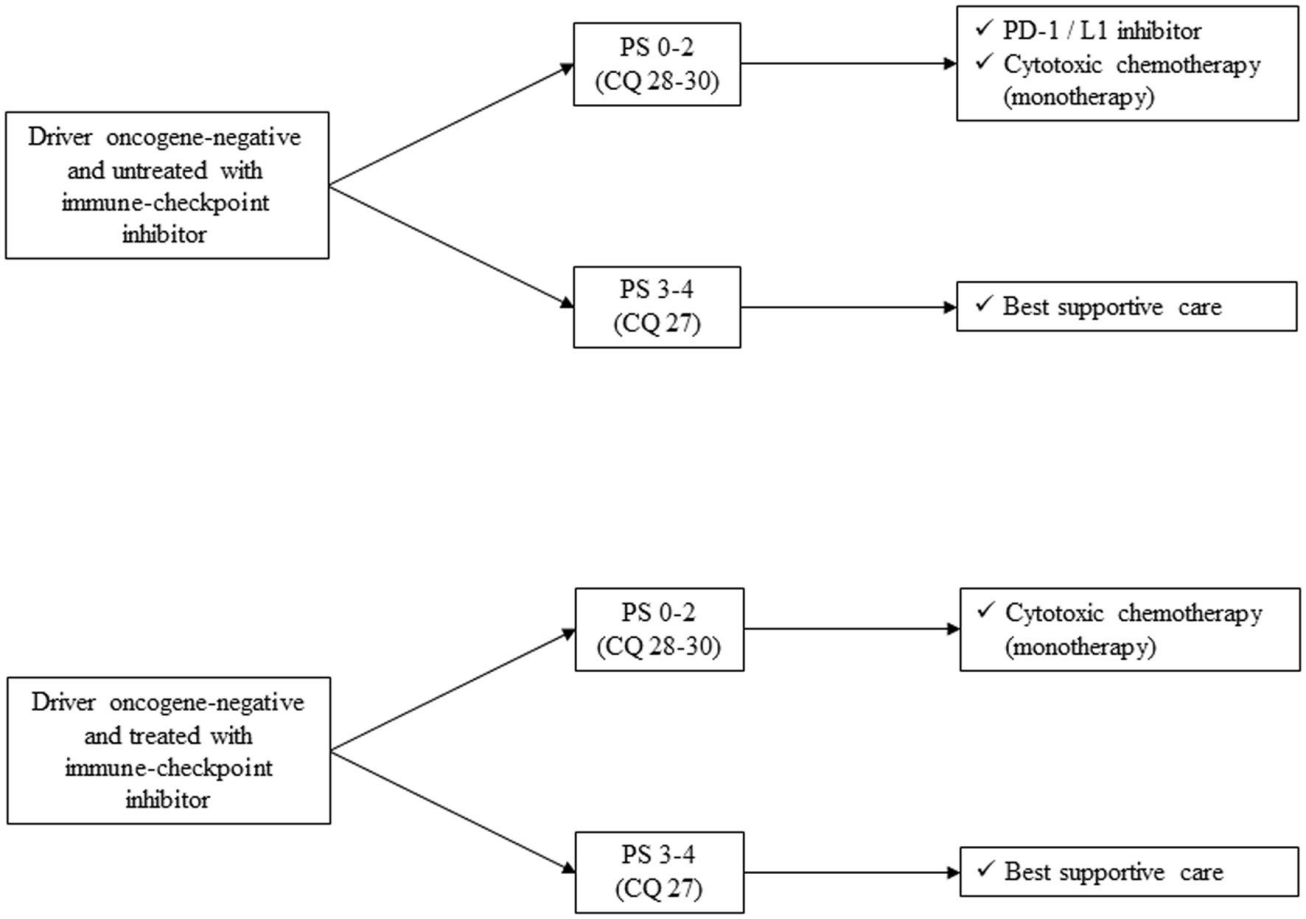
Second-line and further treatment in patients with stage IV NSCLC, who were driver oncogene-negative and PD-L1 was < 50%, or unknown. *PD-1* programed cell death-1, *PD-L1* programed death-ligand 1, *PS* performance status

##### CQ 28

Is chemotherapy recommended as a second- or further-line treatment in patients with PS 0–2 who progressed with first-line treatment other than PD-1/PD-L1 inhibitor?

Recommendation:

- PD-1/PD-L1 inhibitor is strongly recommended.

Recommendation: 1

Evidence level: A

Agreement rate 100%

(b) Cytotoxic chemotherapy is weakly recommended.

Recommendation: 2

Evidence level: A

Agreement rate 96%

Comments:

- Several phase III trials comparing PD-1/PD-L1 inhibitors with DTX in the second-line treatment in driver oncogene-negative patients demonstrated prolongation of OS. Studies with nivolumab or atezolizumab were conducted regardless of PD-L1 expression (CheckMate-017 study, CheckMate-057 study, OAK study [118–120]), while a study of pembrolizumab was conducted only in patients whose tumor expressed PD-L1 > 1% (KEYNOTE-010 study [121]).
  These trials enrolled patients with PS 0–1; thus, efficacy and safety data of PD-1/PD-L1 inhibitor in patients with PS 2 in the second-line setting were unclear. However, considering milder toxicity of these agents compared with cytotoxic drugs, many guideline committee members supported PD-1/PD-L1 inhibitor as an option for this population. On the other hand, ORR of these drugs was 10–20% in the previous reports. Once they are not effective, the guideline committee also recommends a prompt change to cytotoxic chemotherapy.
  Based on this evidence, PD-1/PD-L1 inhibitor is strongly recommended in patients who progressed with the first-line treatment. We judged quality of evidence as A and strength of recommendation as 1.

Involved members regarding this voting: sub-committee on chemotherapy (medical doctors, pharmacists, nurses, statisticians, and patients)

**Table:** 

Benefit with strong recommendation	Benefit with weak recommendation	Unable to determine recommendation	No benefit or risk with weak recommendation	No benefit or risk with strong recommendation
100% (23/23)	0	0	0	0

(b) Of five randomized III trials comparing PD-1/PD-L1 inhibitors with DTX [118–122], several studies showed a cross in Kaplan–Meier curves of PFS or OS. This may suggest that some patients benefited from DTX. In addition, DTX + RAM significantly prolonged OS compared to DTX alone, but this combination has not been compared with any PD-1/PD-L1 inhibitors.
  Based on this evidence, cytotoxic chemotherapy is weakly recommended in patients who progressed with first-line treatment. We judged quality of evidence as A and strength of recommendation as 2.

Involved members regarding this voting: sub-committee on chemotherapy (medical doctors, pharmacists, nurses, statisticians, and patients)

**Table:** 

Benefit with strong recommendation	Benefit with weak recommendation	Unable to determine recommendation	No benefit or risk with weak recommendation	No benefit or risk with strong recommendation
0	96% (22/23)	4% (1/23)	0	0

##### CQ 29

What is the recommended chemotherapy as the second- or further-line treatment in patients with PS 0–2?

Recommendation:

DTX+/-RAM, PEM, or S-1 is strongly recommended.

Recommendation: 1

Evidence level: A

Agreement rate 100%

Comment:

Two phase III trials of DTX in patients who progressed with first-line treatment were reported. In TAX-320 study, DTX 75 mg/m^2^ showed improvement in ORR, PFS rate at 26 weeks, and OS rate at 1 year compared with VNR or IFM, although they were not statistically significant [123]. In another study, comparing DTX 75 mg/m^2^ with BSC, MST and OS rate at 1 year were superior in DTX arm (7.5 months versus 4.6 months, 37% versus 19%, respectively), and QOL was also improved [124]. In a Japanese phase II trial, 60 mg/m^2^ of DTX showed relatively similar efficacy (ORR 18.2% and OS 7.8 months) [125]. Regarding evidence of DTX + RAM, refer to CQ 30.

In a phase III trial comparing PEM with DTX, non-inferiority of OS was not demonstrated [8.3 months versus 7.9 months, HR 0.99 (95%CI: 0.80–1.20)], but seemed similar. Regarding toxicity, neutropenia and febrile neutropenia (grade 3–4) and alopecia (any grade) were increased in DTX arm. In non-squamous cell lung cancer subset, OS was longer in PEM arm [9.3 months versus 8.0 months, HR 0.78 (95%CI 0.61-1.00), *p* = 0.076] [126, 127].

An East Asian phase III trial comparing S-1 with DTX in patients with PS 0–2 as their second- or third-line treatment was conducted [128]. Non-inferiority of OS was demonstrated [12.8 months versus 12.5 months, HR 0.95 (95%CI 0.83–1.07), *p* = 0.38]. ORR and PFS were similar (ORR: 8.3% versus 9.9%, mPFS: 2.9 months in both arms). Regarding toxicity, neutropenia ≥ grade 3 and febrile neutropenia were higher in DTX arm (0.9% versus 13.6% and 5.4% versus 47.7%, respectively), while diarrhea ≥ grade 3 and oral mucositis were higher in S-1 arm (37.2% versus 18.2% and 23.9% versus 14.5%, respectively).

Based on this evidence, DTX+/-RAM, PEM, or S-1 is strongly recommended in patients with PS 0–2 as their second- or further-line treatment. We judged quality of evidence as A and strength of recommendation as 1.

Involved members regarding this voting: sub-committee on chemotherapy (medical doctors, pharmacists, nurses, statisticians, and patients)

**Table:** 

Benefit with strong recommendation	Benefit with weak recommendation	Unable to determine recommendation	No benefit or risk with weak recommendation	No benefit or risk with strong recommendation
100% (23/23)	0	0	0	0

##### CQ 30

Is addition of RAM to DTX recommended in the second-line treatment?

Recommendation:

- Addition of RAM to DTX is weakly recommended in patients with PS 0–1.

Recommendation: 2

Evidence level: B

Agreement rate 74%

(b) Not to add RAM with DTX is weakly recommended in patients ≥ 75 years old.

Recommendation: 2

Evidence level: D

Agreement rate 78%

(c) Not to add RAM with DTX is weakly recommended in patients with PS 2.

Recommendation: 2

Evidence level: D

Agreement rate 87%

Comment:

- PS 0–1
  A phase III trial of DTX + RAM compared with DTX alone in patients who progressed with first-line platinum-doublet chemotherapy was reported (REVEL study) [129]. OS (primary endpoint) was significantly prolonged in DTX + RAM arm [10.5 months versus 9.1 months, HR 0.86 (95%CI 0.75–0.98), *p* = 0.023]. ORR and PFS were also prolonged (ORR: 23% versus 14%, mPFS: 4.5 months versus 3.0 months). Regarding toxicity, neutropenia ≥ grade 3, febrile neutropenia, platelet decreased (any grade), and mucositis were higher in DTX + RAM arm. Hypertension ≥ grade 3 was 6% and most hemorrhagic events were grade 1–2.
  In a Japanese randomized phase II trial (JVCG study), PFS tended to be longer [5.2 months versus 4.2 months, HR 0.83 (95%CI 0.59–1.16)] [130]. Tendency in ORR and OS were similar (ORR: 28.9% versus 18.5%, OS 15.2 months versus 13.9 months). Febrile neutropenia was commonly observed in DTX + RAM arm (34% versus 19%).
  Based on this evidence, addition of RAM to DTX is weakly recommended in patients with PS 0–1. We judged quality of evidence as B and strength of recommendation as 2.
  Involved members regarding this voting: sub-committee on chemotherapy (medical doctors, pharmacists, nurses, statisticians, and patients)

**Table:** 

Benefit with strong recommendation	Benefit with weak recommendation	Unable to determine recommendation	No benefit or risk with weak recommendation	No benefit or risk with strong recommendation
26% (6/23)	74% (17/23)	0	0	0

(b) ≥ 75 years old
  Regarding the elderly patients ≥ 75 years old, there are no data regarding the REVEL study. In JVCG study, only 10 patients were ≥ 75 years old. On the other hand, in terms of safety, addition of bevacizumab is not recommended for the elderly in the first-line treatment.
  Based on this evidence, not to add RAM with DTX is weakly recommended in patients ≥ 75 years old. We judged quality of evidence as D and strength of recommendation as 2.

Involved members regarding this voting: sub-committee on chemotherapy (medical doctors, pharmacists, nurses, statisticians, and patients)

**Table:** 

Benefit with strong recommendation	Benefit with weak recommendation	Unable to determine recommendation	No benefit or risk with weak recommendation	No benefit or risk with strong recommendation
0	9% (2/23)	9% (2/23)	78% (18/23)	4% (1/23)

(c) PS 2
  There are no efficacy data of DTX + RAM, because the REVEL study and JVCG study excluded these patients. Considering the rate of febrile neutropenia in patients with PS 0–1, clinicians should heed concern regarding the safety in patients with PS 2.
  Based on this evidence, not to add RAM with DTX is weakly recommended in patients with PS 2. We judged quality of evidence as D and strength of recommendation as 2.

Involved members regarding this voting: sub-committee on chemotherapy (medical doctors, pharmacists, nurses, statisticians, and patients)

**Table:** 

Benefit with strong recommendation	Benefit with weak recommendation	Unable to determine recommendation	No benefit or risk with weak recommendation	No benefit or risk with strong recommendation
0	0	9% (2/23)	87% (20/23)	4% (1/23)

##### CQ 31

Is erlotinib recommended in the second-line treatment?

Recommendation:

Not to administer erlotinib is weakly recommended in patients who are *EGFR wild-type* or unknown.

Recommendation: 2

Evidence level: C

Agreement rate 65%

Comment:

In a phase III trial comparing erlotinib with BSC in patients who progressed with first-line treatment (BR-21 study), OS (primary endpoint) was significantly prolonged [6.7 months versus 4.7 months, HR 0.70 (95%CI 0.58–0.85), *p* < 0.001], and PFS was also prolonged (mPFS: 2.2 months versus 1.8 months, HR 0.61 (95%CI 0.51–0.74)] [131]. This study included both *EGFR-mutated* and *wild-type* patients.

On the contrary, in another phase III trial, which compared erlotinib with DTX in patients who were *EGFR wild-type* (TAILOR study), OS was significantly prolonged in DTX arm [8.2 months versus 5.4 months, HR 0.73 (95%CI 0.53-1.00), *p* = 0.05] [132]. In Japan, erlotinib was compared with DTX in a phase III trial [133]. In a subset of *EGFR wild-type* patients, PFS was superior in DTX arm [2.9 months versus 1.3 months, HR 1.57 (95%CI 1.18–2.11), *p* < 0.01].

Recently, PD-1/PD-L1 inhibitors and DTX + RAM demonstrated superiority to DTX, and S-1 demonstrated non-inferiority to DTX. Considering this situation, efficacy of erlotinib is relatively low. In addition, those patients who were *EGFR wild-type* had some clinical risk factors of interstitial lung disease with EGFR-TKI.

Based on this evidence, not to administer erlotinib is weakly recommended in patients who are *EGFR wild-type* or unknown. We judged quality of evidence as C and strength of recommendation as 2.

Involved members regarding this voting: sub-committee on chemotherapy (medical doctors, pharmacists, nurses, statisticians, and patients)

**Table:** 

Benefit with strong recommendation	Benefit with weak recommendation	Unable to determine recommendation	No benefit or risk with weak recommendation	No benefit or risk with strong recommendation
0	13% (3/23)	17% (4/23)	65% (15/23)	4% (1/23)
